# Spinal cord abnormal autophagy and mitochondria energy metabolism are modified by swim training in SOD1-G93A mice

**DOI:** 10.1007/s00109-023-02410-8

**Published:** 2024-01-10

**Authors:** Katarzyna Patrycja Dzik, Damian Józef Flis, Katarzyna Barbara Kaczor-Keller, Zofia Kinga Bytowska, Mateusz Jakub Karnia, Wiesław Ziółkowski, Jan Jacek Kaczor

**Affiliations:** 1 Department of Animal and Human Physiology, Faculty of Biology, University of Gdansk, Bazynskiego 8, 80-309 Gdansk, Poland; 2 Department of Pharmaceutical Pathophysiology, Faculty of Pharmacy, Medical University of Gdansk, Gdansk, Poland; 3 Department of Molecular Biology, Institute of Genetics and Animal Biotechnology, Polish Academy of Science, Magdalenka, Poland; 4 Division of Bioenergetics and Physiology of Exercise, Faculty of Health Sciences With Institute of Maritime and Tropical Medicine, Medical University of Gdansk, Gdansk, Poland; 5 Department of Rehabilitation Medicine, Faculty of Health Sciences With Institute of Maritime and Tropical Medicine, Medical University of Gdansk, Gdansk, Poland

**Keywords:** Amyotrophic lateral sclerosis, Exercise, Mitochondria, Autophagy

## Abstract

**Abstract:**

Amyotrophic lateral sclerosis (ALS) may result from the dysfunctions of various mechanisms such as protein accumulation, mitophagy, and biogenesis of mitochondria. The purpose of the study was to evaluate the molecular mechanisms in ALS development and the impact of swim training on these processes. In the present study, an animal model of ALS, SOD1-G93A mice, was used with the wild-type mice as controls. Mice swam five times per week for 30 min. Mice were analyzed before ALS onset (70 days old), at ALS 1 disease onset (116 days old), and at the terminal stage of the disease ALS (130 days old), and compared with the corresponding ALS untrained groups and normalized to the wild-type group. Enzyme activity and protein content were analyzed in the spinal cord homogenates. The results show autophagy disruptions causing accumulation of p62 accompanied by low PGC-1α and IGF-1 content in the spinal cord of SOD1-G93A mice. Swim training triggered a neuroprotective effect, attenuation of NF-l degradation, less accumulated p62, and lower autophagy initiation. The IGF-1 pathway induces pathophysiological adaptation to maintain energy demands through anaerobic metabolism and mitochondrial protection.

**Graphical Abstract:**

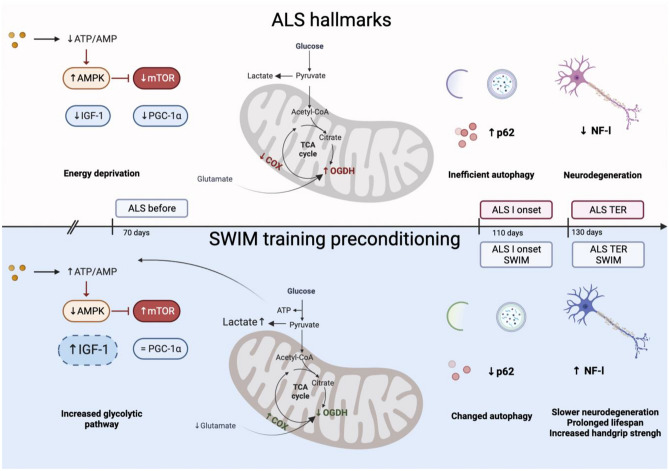

**Key messages:**

- The increased protein content of p62 in the spinal cord of SOD1-G93A mice suggests that autophagic clearance and transportation are disrupted.
- Swim training attenuates neurofilament light destruction in the spinal cord of SOD1-G93A mice.
- Swim training reducing OGDH provokes suppression of ATP-consuming anabolic pathways. Swim training induces energy metabolic changes and mitochondria protection through the IGF-1 signaling pathway.

**Supplementary Information:**

The online version contains supplementary material available at 10.1007/s00109-023-02410-8.

## Introduction

Amyotrophic lateral sclerosis (ALS) is a neurodegenerative disease with no effective treatment. Several major cellular disruptions have been identified, which are involved with ALS pathogenesis, including oxidative stress and mitochondrial damage [1], autophagy dysregulation [2], or accumulation of glutamate receptors, leading to excitotoxicity, and impaired clearance of neurotoxic dipeptide repeat proteins derived from the repeat expansion [3]. All of the above may cause the ALS hallmark, which is the accumulation of misfolded proteins in damaged neurons [4], and lead to cell death [5]. The primary ALS cause remains unknown. It may result from abnormalities in mechanisms, which under normal conditions act to maintain proper organelle homeostasis. Besides protein accumulation, dysregulations of homeostatic mechanisms in ALS include altered axonal transport and mitochondria disruptions. The last one includes not only the biogenesis of mitochondria mediated by the transcription coactivator peroxisome proliferator-activated receptor gamma coactivator 1 alpha (PPARGC1A or PGC-1α) [6], but also the fission/fusion process and mitophagy. Interestingly, in SOD1-G93A mice, the metabolic abnormalities are most pronounced in the spinal cord [7].

The possible signaling molecules embroiled in the ALS progression are insulin-like growth factor 1 (IGF-1) and tank-binding kinase 1 (TBK-1). IGF-1 is a pluripotent growth factor with multiple functions in the peripheral and central nervous system (CNS) [8], including mitochondrial protection [9]. TBK-1, identified as an ALS gene, is known to bind and phosphorylate several proteins involved in the activation of the immunity system and autophagy and p62 (SQSTM1/sequestosome), which has also been implicated in ALS [10]. p62 is a multifunctional protein that serves as a selective autophagy marker. p62’s domains provide a scaffold that directs substrates to autophagosomes and facilitates the autophagic process. Defective autophagy leads to p62 accumulation, and p62 levels are used as a marker for autophagic flux, along with LC3 (microtubule-associated protein light chain 3 (LC3). A lipidated form of LC3, LC3-II, is an autophagosomal marker in mammals [11], which along with p62 are key players in mitophagy [12].

Mitochondria contain two enzymes that depend on copper for their activity: cytochrome *c* oxidase (COX) and a fraction of copper-zinc superoxide dismutase (SOD1). Most diseases associated with mutations in copper-dependent enzymes and copper-handling proteins, including cytochrome c oxidase assembly protein 1 (SCO1) and SCO2, have standard symptomatic features, namely, neuromuscular defects and motor symptoms [13]. SCO1/2 mutations cause mitochondrial complex IV deficiency [14]. Beyond that, the relevance of copper in motor neuron maintenance and, ultimately, neuromuscular degeneration is evidenced by the solid genetic and pathological association of SOD1 with familial ALS. Copper deficiency accelerates aberrant hydrophobicity of both WT and mutant SOD1 due to partial protein unfolding [15, 16]. Another mitochondrial enzyme seems to be critical to properly assess mitochondrial function: 2-oxoglutarate dehydrogenase (OGDH) is not only a regulatory point at the cross-road of the tricarboxylic acid (TCA) cycle for energy production but also for de novo generation of the glutamate neurotransmitter from glucose in the TCA cycle. Indeed, the level of glutamate, the main excitatory neurotransmitter in the CNS, is tightly linked to the brain’s OGDH function [17]. OGDH appears to have a lower activity in the human brain than other TCA cycle enzymes. Therefore, it has a high metabolic control coefficient for overall oxidative energy metabolism. Additionally, the OGDH complex can sense and generate reactive oxygen species (ROS) [18]. Therefore, the measurement of COX, among its subunits COX II (mitochondrially) and COX IV (nuclearly encoded), OGDH, and SCO1/2, seems to be crucial to assess the function of mitochondria under the ALS circumstances.

The study aimed to explore the possible signaling mechanism as a result of swim training responsible for pathophysiological adaptation including the autophagic abilities in the spinal cord of SOD1-G93A mice and to investigate whether changes in the signaling protein content will improve the function of mitochondria and abnormal autophagy.

## Materials and methods

### Experimental model and subject details

Transgenic male mice (B6SJL-TgN[SOD1-G93A]1Gur) and wild-type (WT) male B6SJL mice were purchased from Jackson Laboratory (Bar Harbor, ME, USA). After a 2-week adaptation period, SOD1-G93A mice were randomly assigned to ALS before (*n* = 8), ALS 1 onset (*n* = 8), ALS 1 onset SWIM (*n* = 7), ALS TER (*n* = 10), and ALS TER SWIM (*n* = 10). WT male B6SJ served as the controls. Experimental procedures were performed following European animal research laws (European Communities Council Directive 2010/63/EU). All the procedures were approved by the Local Ethics Committee (resolution number 11/2013) and the Polish Ministry of the Environment (decision number 155/2012). The purchase of the animals was founded by a grant from the Polish National Science Center (DEC-2013/09/N27/02538).

### Housing and euthanasia

Mice were housed in an environmentally controlled room (23 ± 1 °C with a 12-h light–dark cycle, humidity 55%). Mice received standard mouse chow and water ad libitum. All of the mice were euthanized by cervical dislocation. Mice from the ALS before and WT groups were euthanized on the 70th day of life. Mice from the ALS 1 onset group were euthanized at age of 116 ± 2 days of life, when the first symptoms of the disease were observed. Mice from the ALS 1 onset SWIM, WT 1 onset, and WT 1 onset SWIM groups were sacrificed at the same age as the ALS 1 onset group. Mice from the ALS TER group were euthanized when functional paralysis in both hind legs was observed (the terminal stage of the disease, 130 ± 3 days of age). The ALS TER SWIM, WT TER, and WT TER SWIM mice were sacrificed at the same age as the ALS TER mice.

### Swim training protocol

The ALS SWIM 1 and ALS TER SWIM groups underwent swim training according to Deforges et al. [19] with the modification described by Flis et al. [20]. Briefly, the training procedure started at the age of 10 weeks. The training was held five times per week, for 30 min, in the water at a temperature 30 °C, without additional weight. Training was performed in the specially designed swimming pool generating water current with an adjustable flow (max. 5 l min^−1^).

### Sample preparation and material collection

After cervical dislocation, the spinal cords were collected, divided into 2 pieces, quickly frozen on liquid nitrogen, and stored at – 80 °C until further analysis. For the ELISA and enzyme activities measurements, weighted pieces of spinal cords were homogenized in glass homogenizer (4%wt/vol) in lysis buffer (50 mM Tris–HCl, 150 mM NaCl, 1 mM EDTA, 0.5 mM DTT). For the Western Blot (WB) analysis, the tissues were homogenized (8%wt/vol) in the Pierce RIPA buffer (Thermo Scientific™, cat. # 89,901). Both buffer solutions were enriched with EDTA-free Protease Inhibitor Cocktail (Roche, Switzerland, cat. # 04693159001) and PhosSTOP™ phosphatase inhibitors (Roche, Switzerland, cat. # 04906837001). Homogenates for ELISA and enzyme activities measurement were centrifuged at 750 g, and then part of the obtained supernatants was spun at 5000 g for 10 min at 4 °C. For the WB analysis, the homogenates were centrifuged at 15,000 g for 10 min at 4 °C. The supernatant was collected and frozen for further analysis. All samples were coded, and the staff members remained blind for the group assessment of all of the measurements in the spinal cord. Protein concentration was measured with the Bradford method.

### Western blot analysis

Equal amounts of total tissue lysates were resolved in 10–12% SDS–polyacrylamide gel electrophoresis (SDS-PAGE) and transferred onto a polyvinylidene difluoride (PVDF) membrane. The membranes were then blocked with Every Blot Blocking Buffer (Bio-Rad, cat. # 12,010,020) or solution containing 10 mM Tris-buffered saline, 0.05% Tween 20, and 5% nonfat dry milk. Then, membranes were incubated over night at 4 °C with primary antibodies including LC3A/B (Cell Signaling Technology, cat. # 1241S, 1:1000), Beclin-1 (Cell Signaling Technology, cat. # 3495S, 1:1000), p62 (Cell Signaling Technology, cat. # 39749S, 1:1000), AMPK (Cell Signaling Technology, cat. # 5832S, 1:1000), pAMPK (Cell Signaling Technology, cat. # 2537S, 1:1000), mTOR (Cell Signaling Technology, cat. # 2983S, 1:1000), p-mTOR (Cell Signaling Technology, cat. # 5536S, 1:1000), β-Tubulin (Cell Signaling Technology, cat. #, 1:1000), COX IV (Cell Signaling Technology, cat.# 11967S, 1:1000), neurofilament light NF-l; (Cell Signaling Technology, cat. # 2837S, 1:1000), OGDH (NovusBio, cat. # NBP2-19,622, 1: 2000), MT-CO2 (NovusBio, cat. # NBP2-94,364, 1:1000), SCO1 (NovusBio, cat. # NBP1-77,273, 1:1000), SCO2 (NovusBio, cat. # NBP2-94,525, 1:1000), and IGF-1 (Abcam, cat. # ab9572, 1:10,000). For PGC-1α (Abcam, cat. # ab191838, 1:2000), the membranes were blocked over night with 10 mM Tris-buffered saline, 0.05% Tween 20, and 5% nonfat dry milk and then incubated with primary antibodies for 1 h. After the washing procedure (3 × 10 min in 1xTBST), membranes were incubated for 1 h at room temperature and gentle shaking with secondary antibodies: anti‐Goat Anti-Rabbit IgG (H + L)-HRP Conjugate (Bio-Rad, cat. #170–6515, 1:3000) and Goat Anti-Mouse IgG (H + L)-HRP Conjugate (Bio-Rad, cat. #1,706,516, 1:30,000). Following treatment with the appropriate secondary antibody, the bands were visualized using ChemiDoc (GE Healthcare). Changes in protein levels were assessed by densitometry of immunoreactive bands and normalized to the total amount of protein in the samples measured on membranes after transfer and to β-tubulin protein load. ChemiDoc image analysis system (Bio‐Rad Laboratories, Inc) analyzed and quantified the relative protein levels as shown. Then, each result of the ALS groups was normalized to the WT group. The immunoblotting was performed at least two times.

### ELISA measurement

For the measurement of COX IV and COX II, we used the following: mouse cytochrome c oxidase subunit 4 isomers 1, mitochondrial ELISA Kit (COX IV-1) (EIAab Science Corporation, Wuhan, China, cat. # E10129m), Mouse cytochrome c oxidase subunit 2 ELISA Kit (EIAab Science Corporation, Wuhan, China, cat. # E15194m). All measurements were performed according to the manufacturer’s instructions. All of the samples were analyzed in duplicate in a microplate reader Thermo Scientific Multiscan Go (Thermo Fisher Scientific, Vantaa, Finland).

### Enzyme activity assessment

Cytochrome c oxidase (COX) activity was measured spectrophotometrically (Cecil CE9200, Cecil Instruments Limited, Cambridge, UK) in spinal cord homogenates. A 20 μl of reduced cytochrome c was added to the tissue in the 50 mM potassium buffer (pH 7.2) with 1 mM EDTA and 0.05% TRITON. The activity of COX was measured through a direct method, following the decrease in absorbance at 550 nm due to the oxidation of reduced cytochrome c, carried out by the enzyme. The enzyme activity was expressed as nmol/minute/mg of protein.

### Quantification and statistical analysis

Analyses were performed using Statistica v. 13.3 (StatSoft Inc., Tulsa, OK, USA). To verify which statistical test should be used, the normality of distribution and similarity of variances were tested. The differences resulted from disease progression (TIME), and differences between SWIM and untrained groups (TRAINING) were performed using two-way ANOVA. If a difference was detected in these test models, the least significant difference (LSD) post hoc test was used for verification.

To verify the significance of the small, swim training-associated changes (ALS 1 onset vs. ALS 1 onset SWIM and ALS TER vs. ALS TER SWIM), the Student *t*-test or Mann–Whitney *U* test was used. The results were considered statistically significant when *p* < 0.05. The results are expressed as the mean ± standard error (SEM).

## Results

Results from the analysis of the clinical score, body mass, and behavioral tests as well as analysis of skeletal muscles and blood were already published [20–23]. The applied swim training procedure caused an increase in lifespan from 126 in the sedentary mice and 135.5 days in the trained ALS mice. Mice in the SWIM groups maintained significantly higher body mass after the first onset of diseases, as well the disease progression measured with clinical score was lower. Moreover, the swim training ameliorated the loss of hand grip strength and reduced hyperlocomotion during the Open Field Test [24]. Swim training significantly improved the respiratory capacity rate [20], measured in tight muscles, and caused major metabolic changes in skeletal muscle metabolism.

**Fig. 1:**
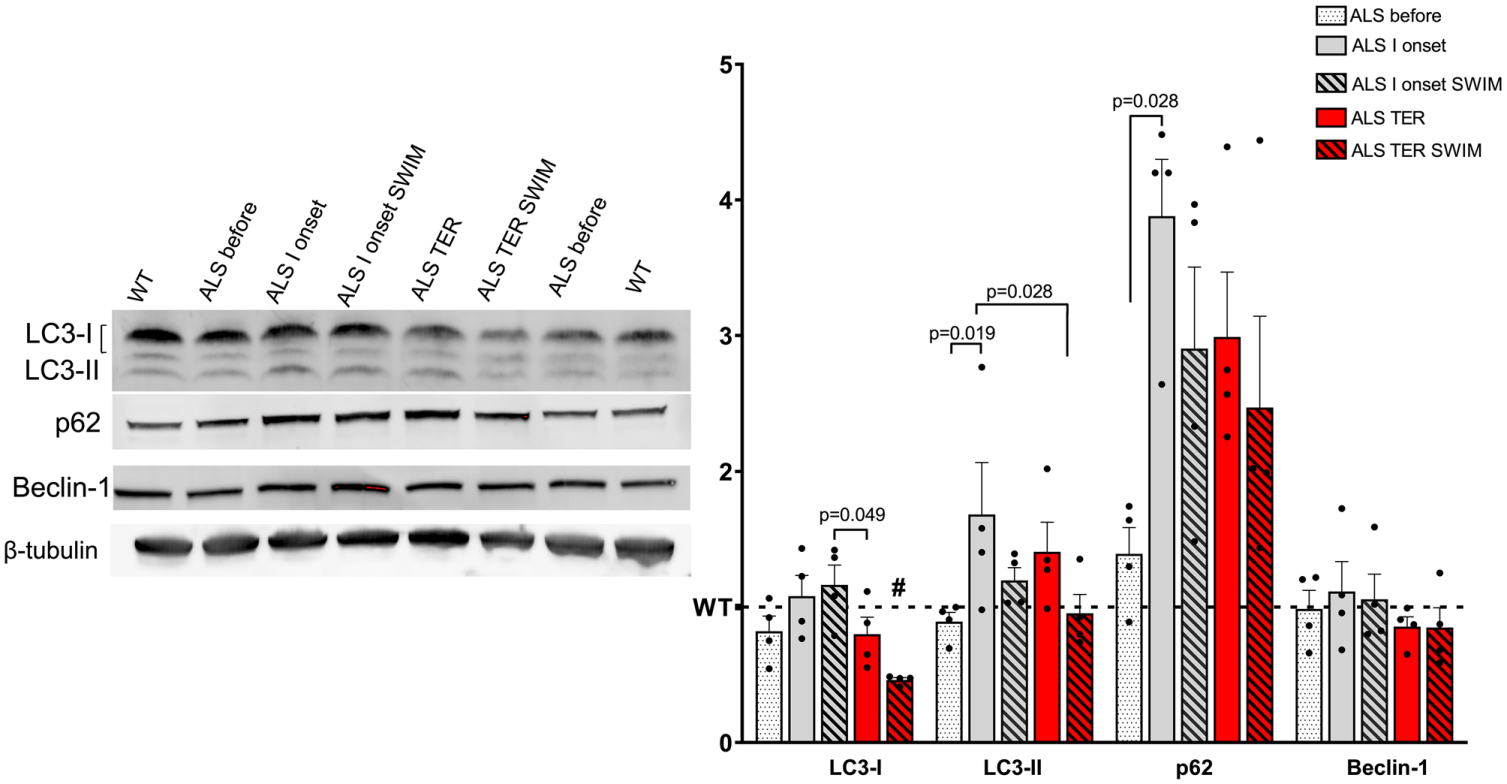
Accumulation of p62 and autophagy disruptions in the spinal cord of SOD1-G93A mice are modified by swim training preconditioning.** A** Western blot of LC3-I/LC3-II, p62, and Beclin-1 content; #*p* = 0.049 versus ALS before, *p* = 0.0025 versus ALS I onset, and *p* = 0.001 versus ALS I onset SWIM. All data are presented as mean ± SEM. The results are normalized to the total protein content. *P* values were obtained using two-way ANOVA followed by LSD post hoc test for multiple comparisons. For ALS before *n* = 4, ALS 1 onset *n* = 4, ALS 1 onset SWIM *n* = 4, ALS TER *n* = 4, and ALS TER SWIM *n* = 4

This study presents significant abnormalities in the spinal cord autophagy markers (Fig. 1). We found increased autophagy (time effect: *F* = 16.55, *p* = 0.001) activity at the first onset of the disease, for the protein content of LC3-I increased in the ALS I onset group compared to the ALS before group (Fig. 1). Surprisingly, at the terminal stage of the disease, we observed a declining trend in LC3-I and LC3-II protein content in the ALS TER group. The LC3-II, the lipidated form of LC3-I, was significantly higher in the ALS I onset group compared to the ALS before group. Swim training significantly decreased the content of LC3-I at the terminal stage of the disease compared to ALS before, ALS I onset, and ALS I onset SWIM groups. In the ALS TER group, LC3-1 and LC3-2 content was 72 and 47% higher than in the corresponding ALS TER SWIM group. The content of p62 in the spinal cord of ALS mice was increased already at the pre-symptomatic stage of the disease compared with the WT group. Starting at the first onset of the disease, p62 significantly elevated regardless of the training intervention compared to the ALS before group. The p62 content was 2.8 times higher in the ALS I onset group than in the ALS before group. Swim training reduced the p62 protein content at the first onset of the disease; the trend was aggravated in the terminal-trained mice. We observed no difference in the content of Beclin-1.

**Fig. 2:**
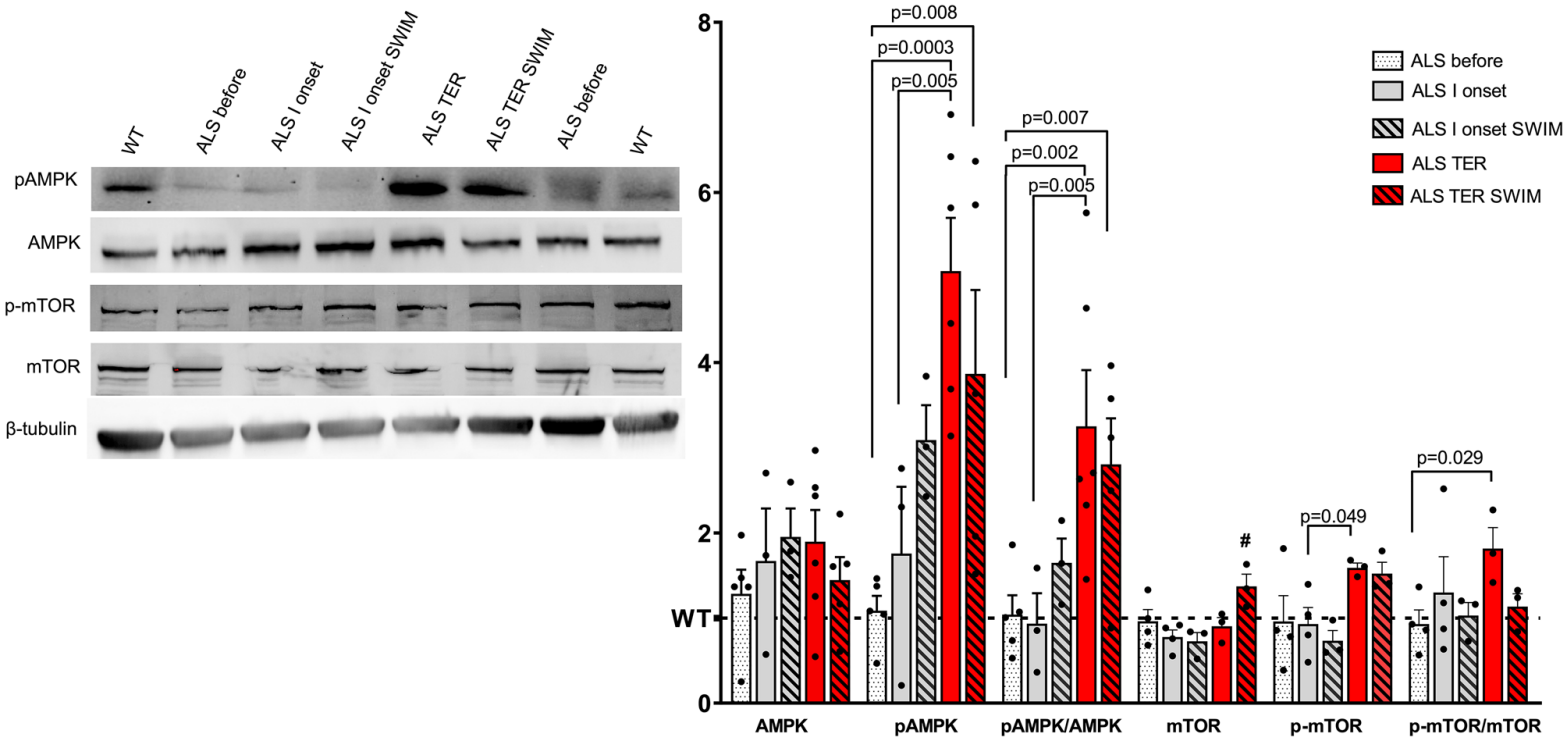
AMPK and mTOR signaling in the spinal cord of SOD1-G93A mice is altered by swim training preconditioning. Western blot of AMPK, pAMPK, mTOR, and p-mTOR content; #*p* = 0.014 versus ALS before group, *p* = 0.009 versus ALS I onset group, *p* = 0.0007 versus ALS I SWIM group, and *p* = 0.004 versus ALS TER group. All data are presented as mean ± SEM. Western blot results are normalized to the total protein content. *P* values were obtained using two-way ANOVA followed by LSD post hoc test for multiple comparisons. For AMPK and pAMPK ALS before *n* = 5, ALS 1 onset *n* = 3, ALS 1 onset SWIM *n* = 3, ALS TER *n* = 6, and ALS TER SWIM *n* = 6. For mTOR and p-mTOR ALS before *n* = 4, ALS 1 onset *n* = 4, ALS 1 onset SWIM *n* = 3, ALS TER *n* = 3, and ALS TER SWIM *n* = 3

The changes in autophagy markers were associated with alterations in AMPK and mTOR phosphorylation states (Fig. 2). The content of AMPK protein was already elevated at the pre-symptomatic stage of the disease in the ALS before, when compared to the WT group. It gradually rose until the terminal stage. The phosphorylated form of AMPK (pAMPK) significantly increased with the disease progression as well; in the ALS TER group, the content of pAMPK was raised more prominently than AMPK (time effect: *F* = 7.55, *p* = 0.014). In the trained groups of mice, the content of both AMPK and pAMPK increased at the first onset of the disease but then dropped at the terminal stage when compared to the corresponding untrained groups. The mTOR content showed the opposite trend. At the I onset of the disease, the content of mTOR dropped (time effect: *F* = 12.2, *p* = 0.003). Swim training caused a significant increase in the mTOR protein in the ALS TER SWIM group compared to the other ALS groups (training effect: 6.03, *p* = 0.026). The phosphorylated form of mTOR (p-mTOR) remained at the level of the WT group in the ALS before, ALS I onset, and ALS I onset SWIM groups. p-mTOR increased at the terminal stage of the disease in both trained and untrained ALS groups (time effect: 5.86, *p* = 0.029).

**Fig. 3:**
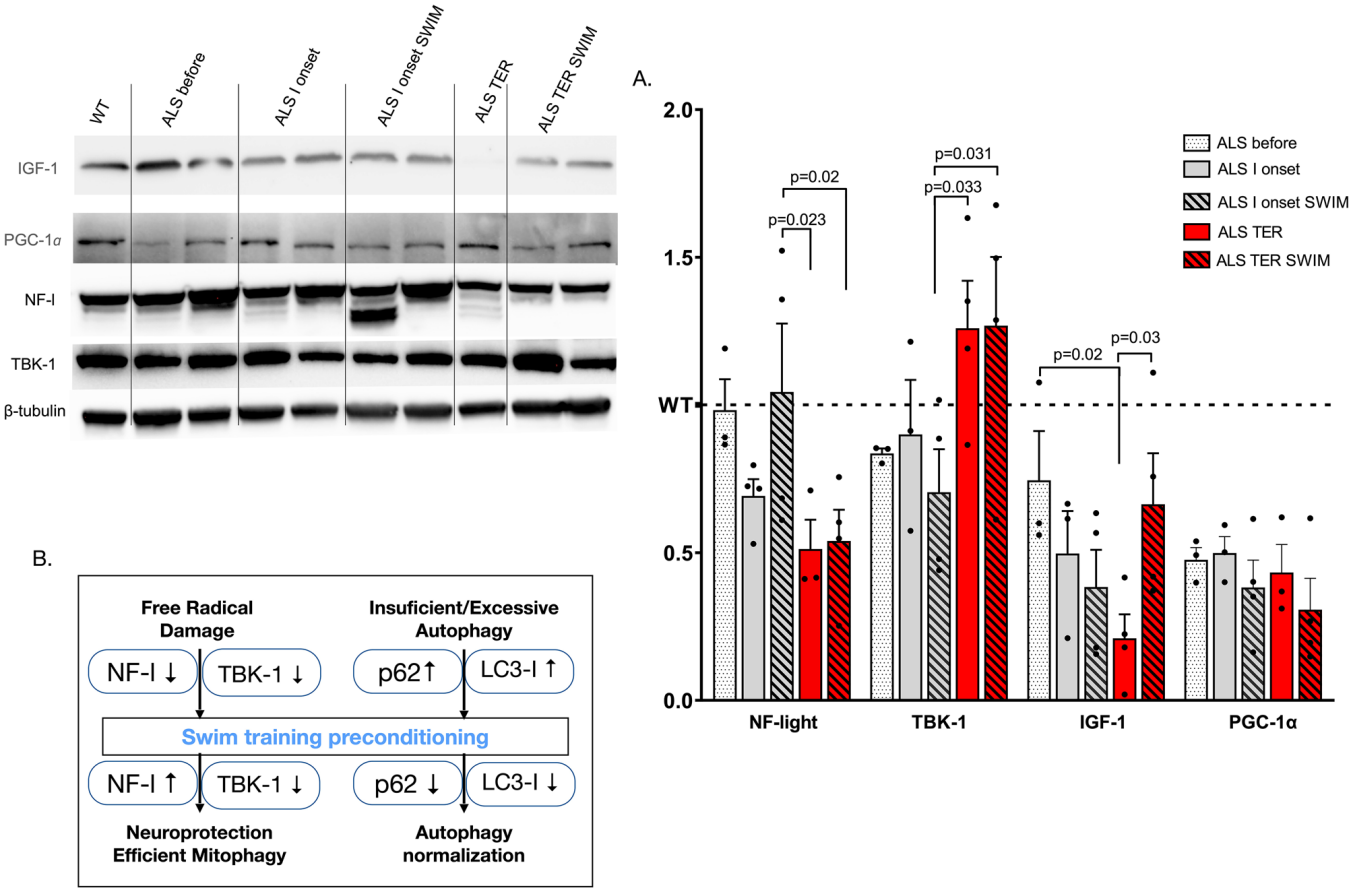
Proteins involved in the signaling pathway in the spinal cord of SOD1-G93A mice. **A** Western blot of NF-l, TBK-1, IGF-1, and PGC-1α content. **B** Hypothesized model of autophagy modifications. All data are presented as mean ± SEM. The results are normalized to the total protein content. *P* values were obtained using two-way ANOVA followed by LSD post hoc test for multiple comparisons. For NF-l: ALS before *n* = 3, ALS 1 onset *n* = 4, ALS 1 onset SWIM *n* = 4, ALS TER *n* = 3, and ALS TER SWIM *n* = 4. For TBK-1: ALS before *n* = 3, ALS 1 onset *n* = 3, ALS 1 onset SWIM *n* = 4, ALS TER *n* = 4, and ALS TER SWIM *n* = 4. For IGF-1: ALS before *n* = 3, ALS 1 onset *n* = 3, ALS 1 onset SWIM *n* = 4, ALS TER *n* = 3, and ALS TER SWIM *n* = 4. For PGC-1α: ALS before *n* = 3, ALS 1 onset *n* = 3, ALS 1 onset SWIM *n* = 4, ALS TER *n* = 4, and ALS TER SWIM *n* = 4

Figure 3 represents the results of other signaling proteins. We noticed a decreased NF-l protein content in the ALS I ONSET and ALS TER groups (time effect:* F* = 5.9, *p* = 0.029). Swim training caused the attenuation of NF-l but only in the ALS I ONSET SWIM group; at the terminal stage of the disease, NF-l protein content significantly decreases. The content of TBK-1 showed the opposite effect (time effect: *F* = 7.23, *p* = 0.019). First, in the ALS I onset group, it is slightly elevated and significantly increases in the ALS TER group. Swim training slightly reduced the TBK-1 content at the I onset of the disease.

Moreover, all ALS groups had a significantly lower protein content of PGC-1α in the spinal cord than the WT group. A similar observation to the low PGC-1α content relates to lower IGF-1 protein content in the spinal cord of ALS mice, observed already at the pre-symptomatic stage of the disease. Compared to the WT group, IGF-1 content was slightly reduced in the ALS before the group and continued to decline with the progression of the disease. Swim training significantly elevated IGF-1 content in the ALS TER SWIM group compared to the untrained corresponding ALS group (training effect: *F* = 4.24, *p* = 0.029), but did not change the content of PGC-1α.

**Fig. 4:**
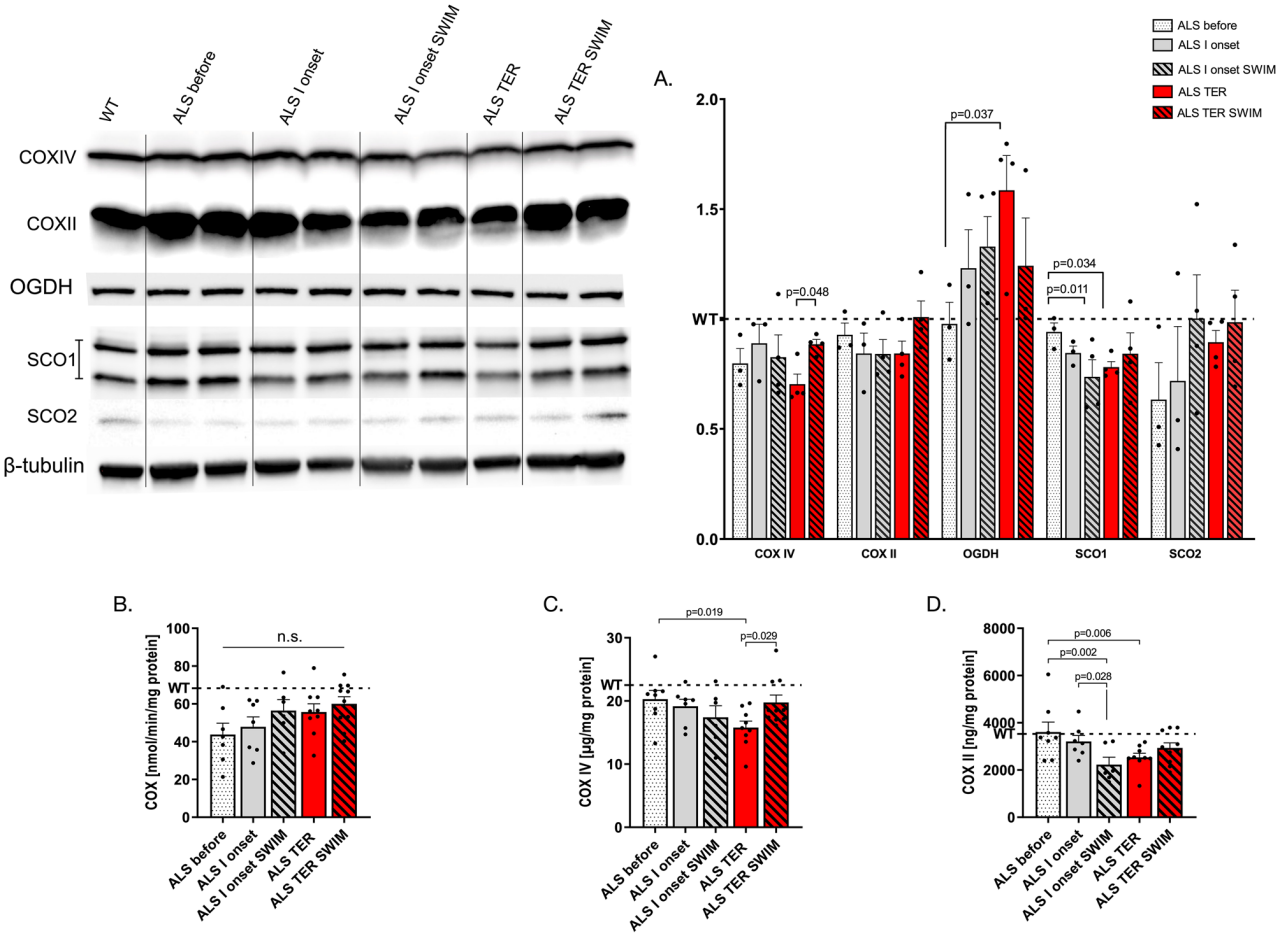
Mitochondrial disruption in the spinal cord of SOD1-G93A mice. **A** Western blot measurement of COX IV, COX II, OGDH, SCO1, and SCO2. **B** The activity of COX. **C** ELISA measurement of COX IV subunit. **D** ELISA measurement of COX II subunit. All data are presented as mean ± SEM. *P* values were obtained using two-way ANOVA followed by LSD post hoc test for multiple comparisons. For **A**: ALS before *n* = 3, ALS 1 onset *n* = 3, ALS 1 onset SWIM *n* = 4, ALS TER *n* = 4, and ALS TER SWIM *n* = 4. For **B**: WT *n* = 8, ALS before *n* = 7, ALS 1 onset *n* = 7, ALS 1 onset SWIM *n* = 6, ALS TER *n* = 9, and ALS TER SWIM *n* = 10. For **C**: WT *n* = 6, ALS before *n* = 8, ALS 1 onset *n* = 7, ALS 1 onset SWIM *n* = 6, ALS TER *n* = 9, and ALS TER SWIM *n* = 10. For **D**: WT *n* = 6, ALS before *n* = 8, ALS 1 onset *n* = 7, ALS 1 onset SWIM *n* = 6, ALS TER *n* = 9, and ALS TER SWIM *n* = 10

Our results showed that the COX activity and COX II and COX IV subunits were lower in the ALS groups in relation to the WT mice (Fig. 4). Surprisingly, COX activity slightly rose in the ALS I onset and ALS TER groups. SWIM training did not significantly influence COX activity. The ALS TER SWIM group also increased COX subunits II and IV. The encoded by nuclear DNA COX IV, which is statistically elevated in the ALS TER SWIM group compared to the corresponding untrained group (training effect: *F* = 3.16, *p* = 0.049), seems more responsive to protecting signaling of swim training than the COX II, encoded by mitochondria. We found OGDH protein content, starting from the first onset of the disease and significantly increasing at the terminal stage of ALS. Swim training did not significantly alter the content of this protein but only slightly reduced its content. Finally, we detected slightly lower SCO1/2 content in the ALS groups compared to the WT group. Interestingly, with disease progression, SCO1 tends to decrease, while SCO2 tends to ameliorate. Swim training increases SCO1 and SCO2 at the terminal stage of the disease.

## Discussion

### Autophagy disruptions cause the accumulation of p62

The increase and decrease in autophagy flux might harm the organism. The observed changes in LC3-I and LC3-II among ALS groups in different stages of the disease show significant disturbances of autophagy flux. The significant increase of the LC3-II content in ALS I onset group compared to the ALS before group suggests abundant autophagosome formation. Nonetheless, the simultaneous increase of p62 shows impaired clearance, indicating blockage of autophagy flux [25]. The declining trend in both LC3-I and LC3-II protein content in the ALS groups at the terminal stage of the disease might indicate that autophagy is most intensely initiated during the development of neurodegeneration. In contrast, during the terminal stage, it appears that we are confronted with the pathophysiological withdrawal of autophagy initiation, most likely attributable to the increased activation of mTOR observed in the ALS TER group. This finding is consistent with previous studies where increased content of p62 was detected in intraneuronal protein aggregates in ALS with dementia patients [26], in oligodendroglial inclusions in the motor cortex of patients with ALS phenotypes caused by mutations in CHMP2B [27] and in other ALS cases [28]. Previously published results from western blotting and immunostaining on the same animal model of ALS demonstrated that p62 is accumulated in parallel with the elevation of polyubiquitinated proteins in the spinal cords and sciatic nerves of SOD1-G93A transgenic mice starting at the early pre-symptomatic stage. The results show that p62 accumulated in protein aggregates in SOD1-G93A transgenic mice long before the disease onset, suggesting that p62 may be involved in the mutant SOD1-mediated ALS etiology [29]. p62 long half-life makes it harder to appreciate significant differences with the short exposures to lysosomal inhibitors. However, these features make p62 an optimal marker to assay any enhancement in the rate of degradation in our neuronal system after long-term treatment with an inducer of autophagy [30].

Intriguingly, both the suppression of p62 [31] and an overexpression in p62 [32] can invalidate ubiquitination-proteasome system function. Both a decrease and an increase in p62 have been associated with the acceleration of disease onset and shortened lifespan in ALS mice [33, 34]. In this study, swim training decreased p62 and at the same time reversed the AMPK and mTOR signaling. Considering that loss of p62 causes aberration in the function of mitochondria but is not required for mitochondrial clearance [35], we decided to investigate the TBK-1 protein content. TBK-1 plays a crucial role in autophagy, including the phosphorylation of several autophagy adaptors, including p62 [36], enhancing their ability to link LC3-II and ubiquitinated cargo [37], therefore enhancing mitophagy [38]. A lower content of TBK-1 at the early stage of ALS would result in impaired mitophagy and accumulation of defective mitochondria, which may contribute to ALS by disrupting axonal transport that occurs in ALS [39]. Surprisingly, the TBK-1 protein content was rising at the terminal stage of the disease despite the training intervention. The observed increases of TBK-1 at the terminal stage might suggest more robust mitochondrial clearance. Our data showed ameliorated 8-isoprostanes and 8-OH guanosine, markers of lipid peroxidation, and DNA damage, respectively, at the terminal stage of the disease, clearly indicating the devastating effects of oxidative stress in the spina3l cord of SOD1-G93A mice [24], what might be the associated with mitochondrial damage. Therefore, the fluctuations of TBK-1 protein content imply ROS-induced mitophagy activation at the terminal stage of the disease. NF-l released into the cerebrospinal fluid as a consequence of axonal damage or degeneration has become the subject of research as a nonspecific marker of neuroaxonal pathology. In ALS, the elevation of NF-l in cerebrospinal fluid levels exceeds that observed in most other neurological diseases, making it useful for the discrimination from mimic conditions and potentially worthy of consideration for introduction as a biomarker for ALS progression [40]. A recent study showed that NF-l is not only a structural protein forming neurofilaments and filling the axonal cytoplasm but also supports the role of NF-l in the regulation of synaptic transmission and organelle trafficking [41]. In the current study, a decrease of NF-l in the spinal cord, deepening with the ALS progression, indicates the development of neurodegeneration. Swim training withholds the loss of NF-l. The increased NF-l content in the ALS I ONSET SWIM group was accompanied by the decreased TBK-1. Therefore, in our opinion, swim training’s impact on the NF-l and TBK-1 suggests that less organelles were damaged, including mitochondria, and consequently, the neuroprotective effect of the intervention was applied.

### ALS causes the attenuation of IGF-1 and PGC-1α signaling in the spinal cord of SOD1-G93A mice

As observed here, low PGC-1α content in the spinal cord of ALS mice is consistent with previous reports where PGC-1α expression was reduced in the motor cortex and spinal cord motor neurons of ALS patients [42], as well as in the spinal cord of the SOD1-G93A mice [42]. For the above, deficiency of PGC-1α seems to be a proper therapeutic target for ALS disease. However, previous studies in the engaging overexpression of PGC-1α in SOD1-G93A mice failed to show any delay in disease onset or progression. Although increasing PGC-1α activity in the muscles of SOD1 mutant-expressing mice produces significantly increased muscle endurance, reduced atrophy, and improved locomotor activity, even at the late stages of the disease, it failed to extend survival [43]. To our surprise, swim training did not alter PGC-1α protein content in the spinal cord, suggesting PGC-1α unrelated coping mechanisms causing the adaptation.

A similar observation to the low PGC-1α content relates to significantly lower IGF-1 protein content in the spinal cord of ALS mice, observed already at the pre-symptomatic stage of the disease. IGF-1, for its neuromodulating effects, is widely investigated regarding a neurodegenerative disorder. Our study is in line with other reports showing the loss of IGF-1 in the brain during aging might contribute to the age-associated decline in cognitive functions [44]. Previous studies showed reduced IGF-1 level in ALS patients’ plasma, indicating that the growth hormone/IGF-1 axis could be a serological marker of some specific neuronal degeneration [45]. Low IGF-1 blood concentrations might play a role in the course of ALS disease by accelerating neurodegenerative changes and worsening clinical symptoms [46]. Presented here, increased IGF-1 content in the ALS trained group seems to trigger a coping mechanism for metabolic adaptation through physical activity. To our knowledge, no reports are showing the impact of IGF-1 elevation in the spinal cord of ALS mice, nonetheless, increasing muscular IGF-1 was found protective in mouse models of ALS [47], and delivering IGF-1 in the CNS also led to ameliorated survival [48, 49]. However, the latest reports showed that the subcutaneous IGF-1 treatment is not beneficial for patients in a 2-year ALS trial. Several reasons have been discussed for the failure of the large trials with subcutaneous administration of IGF-1, in particular, problems with bioavailability or lack of knowledge on how IGF-1 interacts with specific cellular pathways that are dysfunctional in ALS. Therefore, the sole action of IGF-1 requires other metabolic changes to trigger protection in ALS.

### Swim training leads to IGF-1-induced metabolic changes, which downregulate autophagy and protect mitochondria in the spinal cord of SOD1-G93A mice at the terminal stage of the disease

In the ALS TER SWIM group, several phenomena happen at the same time. First, it was already reported that there is a metabolic shift towards anaerobic changes not only in the spinal cord tissue [24] but also in the skeletal muscle and blood of the same mice [23]. In both cases, lactate dehydrogenase (LDH) subunit M4 (enzyme activity in the presence of 2.1 mM pyruvate) was significantly elevated compared to the corresponding group not subjected to the training. Secondly, at the terminal stage of the disease, after the swim training, we found a higher protein content of IGF-1 in the spinal cord, which, along with insulin, are nutrient-sensitive pathways and play a critical role in energy metabolism by regulating glucose metabolism in the CNS [50–53].

The possible explanation for the immense increase of IGF-1 might be related to decreased AMPK and the elevated mTOR content in this group. pAMPK is also decreased in the ALS TER SWIM group, which might imply nutrient-rich conditions [54] Lower pAMPK/AMKP ratio gives less signal for catabolic machinery to trigger the elevates of ATP, which also suggests better metabolic efficiency in the trained mice.

Simultaneously, the mTOR was significantly elevated in the ALS TER SWIM group, possibly due to low AMPK protein content. It seems that elevated LDH activity, low AMPK, high mTOR, and IGF-1 levels are linked closely together and cause the third phenomenon, namely, the reduction of autophagy. Since autophagy is the primary catabolic program of the cell that promotes survival (nutrient delivery) in response to metabolic stress, the observed downregulation of LC3-II, LC3-I, and p62 in the ALS TER SWIM group as compared to the ALS TER group might reflect the coverage of energetic demands through the enhancement of anaerobic abilities via IGF-1 signaling. It seems that the decreased protein content of AMPK results from the increased IGF-1. However, the ability to activate AMPK is closely related to disease progression and the attempt to increase anaerobic energy metabolism. Lastly, at the same time, slight changes in mitochondria function indicate that elevating the protein content of IGF-1 is partially involved in the protection of mitochondria. A recent study on the SOD1-G93A mice in which the scAAV9-hIGF-1 was intramuscularly injected into transgenic cells and administered to cell lines expressing the ∼ 25-kDa C-terminal fragment of transactive response DNA-binding protein (TDP-25) states that IGF-1 vigorously protects mitochondria from apoptosis and upregulates mitophagy in mouse and cell models of ALS [9].

### Swim training diminishes mitochondrial disruption at the terminal stage of ALS

Presented here, increased enzyme activity and protein content of mitochondria in the ALS trained mice is associated with a higher IGF-1 protein content. IGF-1 works by a series of phosphorylation reactions that transduce the signal into the nucleus [55]. Nonetheless, other mitochondrial factors might have been involved in this process. Detected here, tendencies of lowering SCO1/2 content in the ALS groups in relation to the WT group were ameliorated by swim training. In vitro studies have repeatedly indicated that recombinant SOD1 mutants are copper deficient [13, 56]. In the presence of copper, SCO1/2 lead to the metalation and activation of the COX through transferring copper to its catalytic core (subunits I and II), responsible for the mitochondrial electron transfer chain [57, 58]. Therefore, its upregulation might positively affect the oxidative energy metabolism in the spinal cord of ALS trained mice, which is consistent with rises in COX activity in the trained groups.

Surprisingly, despite higher COX II, COX IV, SCO1, and SCO2 content, and slightly increased COX activity, we failed to observe any changes in the PGC-1α content after the swim training application, but the accumulating trend for OGDH content was reversed. Described here, OGDH reduction induced by swim training may cause activation of the intracellular energy sensor, AMPK as a response to oxidative stress in the spinal cord at the terminal stage of ALS [24]. Therefore, activation of AMPK provokes compensatory maintenance in cellular ATP supply by suppressing ATP-consuming anabolic pathways and by activating alternative ATP-producing metabolic pathways by glycolysis [24] or other signaling molecules. Moreover, based on our previous study, which showed elevated markers of lipid and DNA oxidative damage in the ALS TERM group, we assume that a massively increased protein content OGDH is associated with ROS generation by this complex. We are far from any speculation,however, the lowering of OGDH protein content and reduced markers of free radical damage of lipid and DNA in the spinal cord after swim training implies attenuated ROS generation by mitochondria. Besides, our findings of higher COX activity and increased content of subunits II and IV in the ALS TER SWIM group partially confirm the restoration of mitochondria function after swim training at the terminal stage of the disease. It is important to mention that we previously showed significantly higher citrate synthase (CS) in the spinal cord [24] and CS and COX activities in skeletal muscle in the ALS TER SWIM group as compared to the ALS TER group [20]. Moreover, the respiratory capacity ratio, assessed in skeletal muscle crude mitochondria, in the trained mice was 100% higher than in the untrained mice [20]. The data suggest that the upregulation of OGDH might facilitate the forward TCA cycle to satisfy the requirement for the rapid growth of gastric cancer cells [17]. Other studies on hind and forelimb muscles of a SOD1-G93A mouse model also showed elevated OGDH content [59] despite a general reduction in oxidative metabolism, indicating malfunction of mitochondria. It might be related to the increased glutamate content widely described to cause exocitosis in the ALS model.

## Conclusions

ALS disease progression is associated with autophagy disruptions and the accumulation of protein content of p62 in the spinal cord of SOD1-G93A mice. Swim training triggers a neuroprotective effect, attenuation of NF-l degradation, less accumulated p62, and lower autophagy initiation. The IGF-1 pathway induces pathophysiological adaptation of maintaining energy demands through anaerobic metabolism and mitochondrial protection indicated by increased enzymatic activity. Swim training decreasing OGDH provokes compensatory maintenance in cellular ATP supply by suppressing ATP-consuming anabolic pathways. The fluctuations of TBK-1 protein content may suggest ROS-induced mitophagy activation at the terminal stage of the disease in ALS. 

### Supplementary Information

Below is the link to the electronic supplementary material.

Supplementary file1 (PDF 2.14 MB)

Supplementary file2 (PDF 2.70 MB)

Supplementary file3 (PDF 2.62 MB)

Supplementary file4 (PDF 4.57 MB)

## Data Availability

Data will be made available on reasonable request.
