# Supplementary material for: Spinal cord abnormal autophagy and mitochondria energy metabolism are modified by swim training in SOD1-G93A mice
Source: J Mol Med (Berl). 2024 Jan 10;102(3):379–90. doi: 10.1007/s00109-023-02410-8 (PMC10879285; doi:10.1007/s00109-023-02410-8)

Supplementary data.
Full unedited versions of the western blots for Figure 2.

AMPK

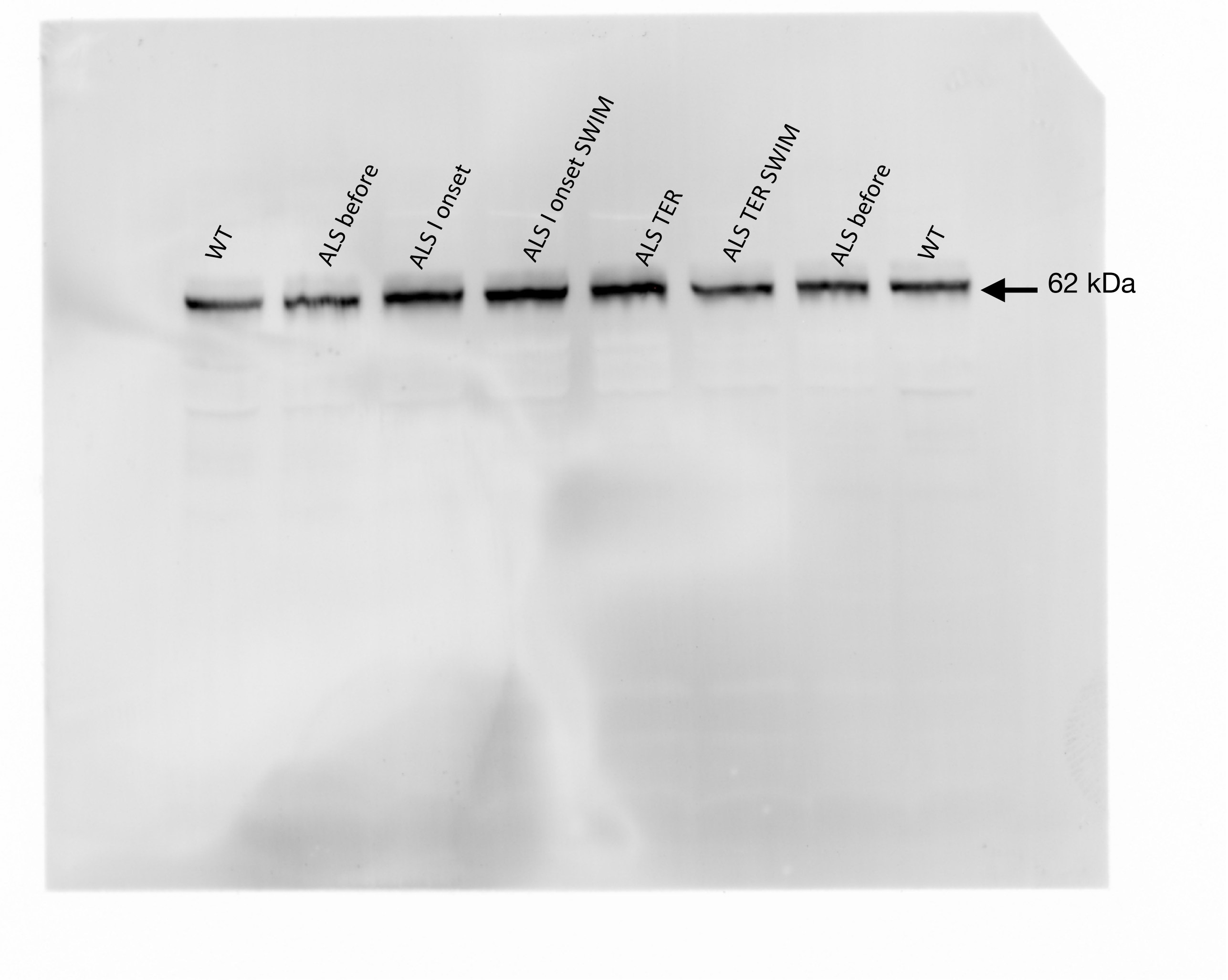

Stain free + protein marker for AMPK

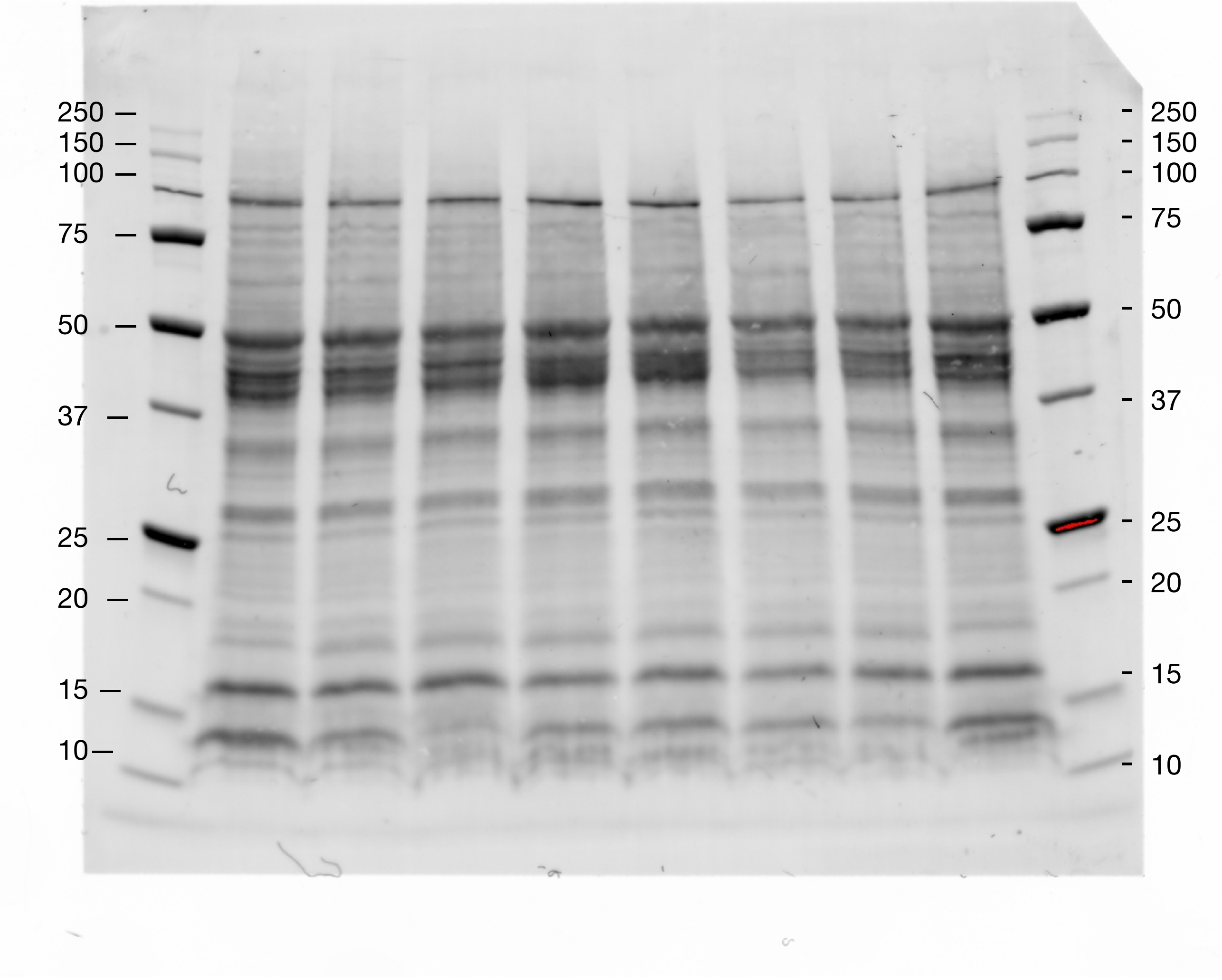

p-AMPK

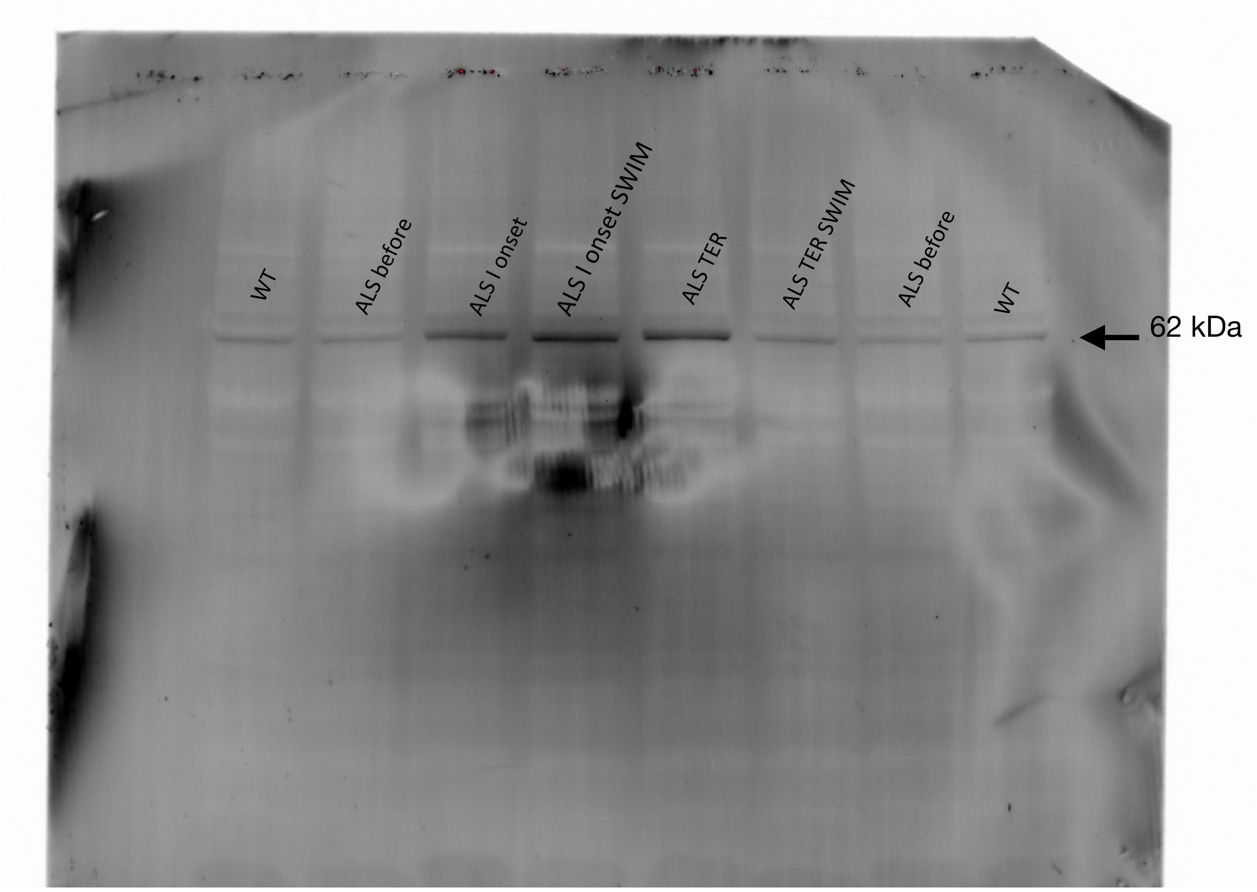

WT
ALS before
ALS I onset
ALS I onset SWIM
ALS TER
ALS TER SWIM
ALS before
WT
62 kDa

Stain free + protein marker for pAMPK

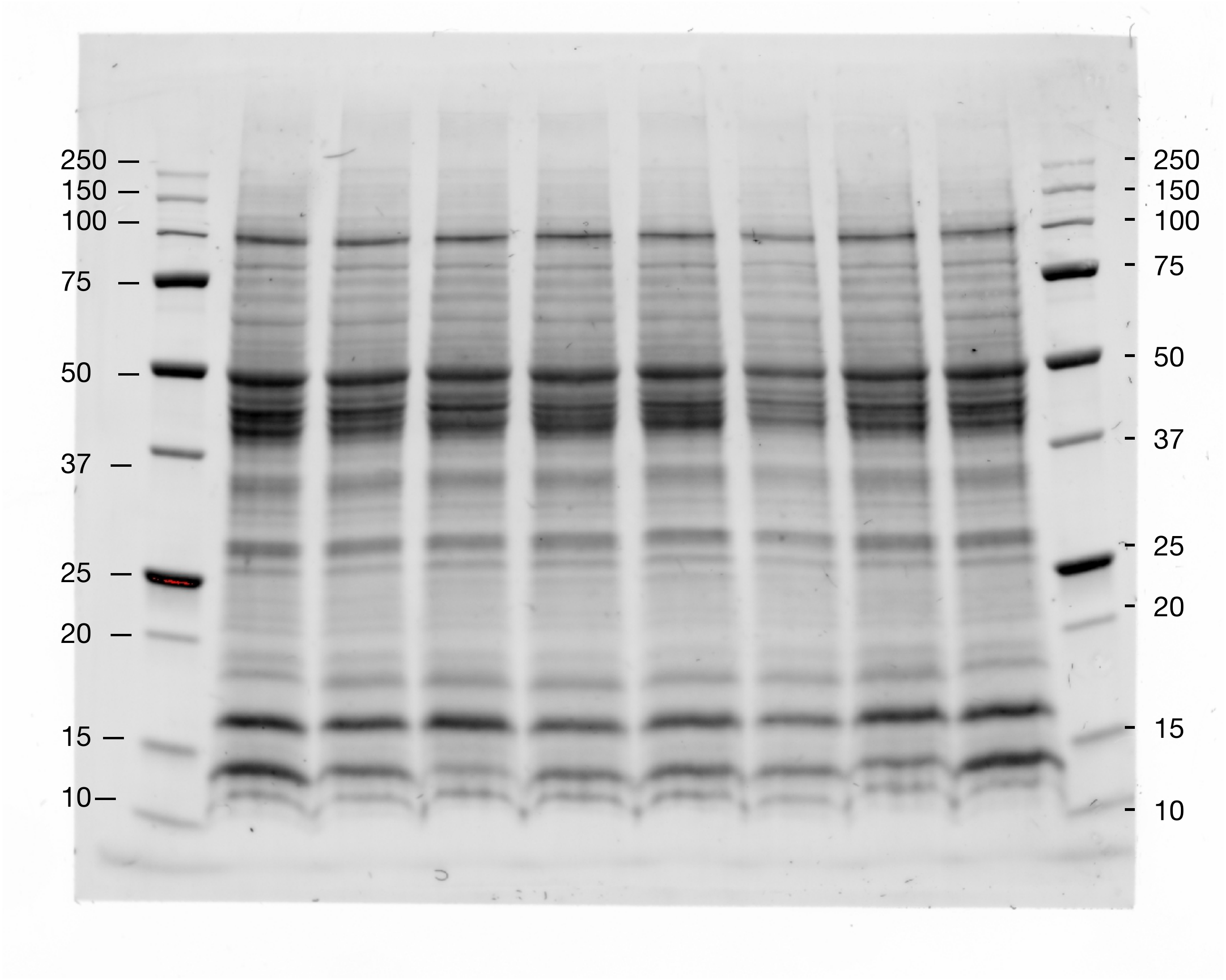

mTOR, membrane cut from LC3 I/II

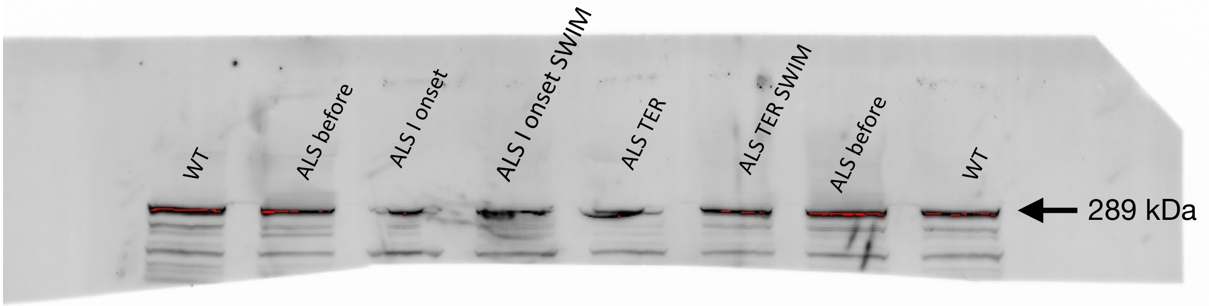

Stain free + protein marker for mTOR

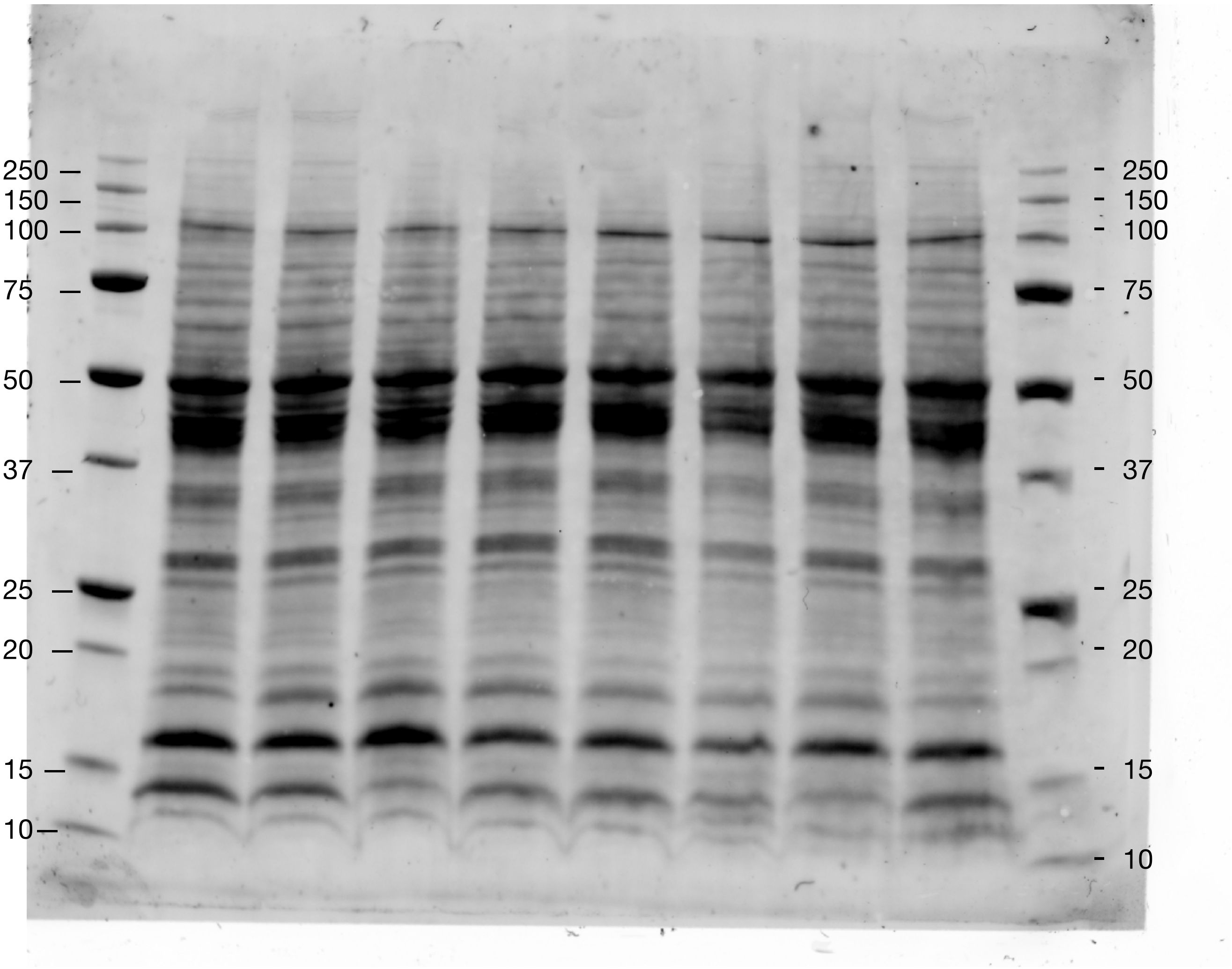

p-mTOR, 289 kDa, membrane cut from p62

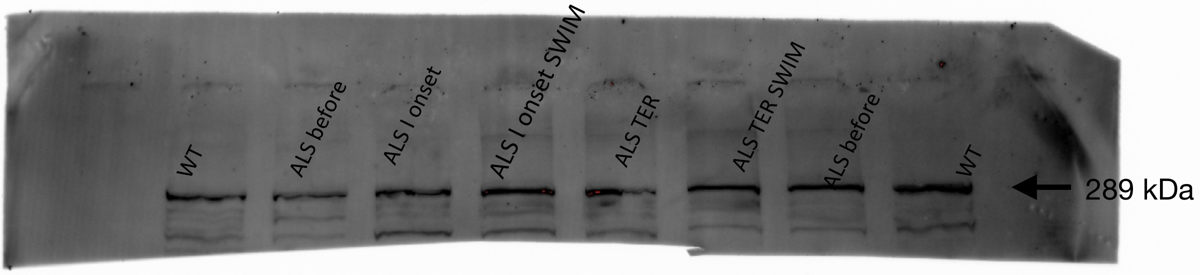

Stain free + protein marker for p-mTOR

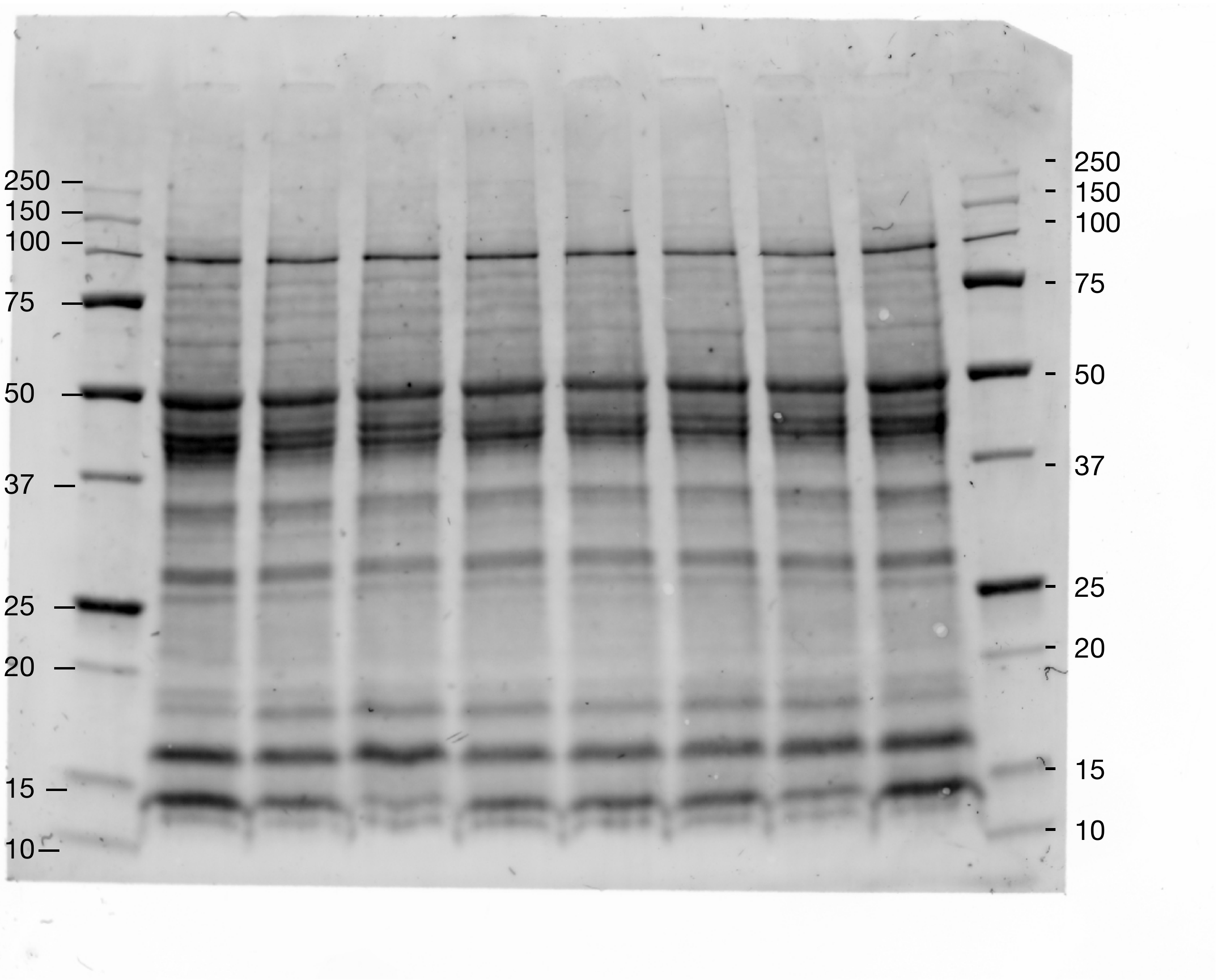

β- tubulin

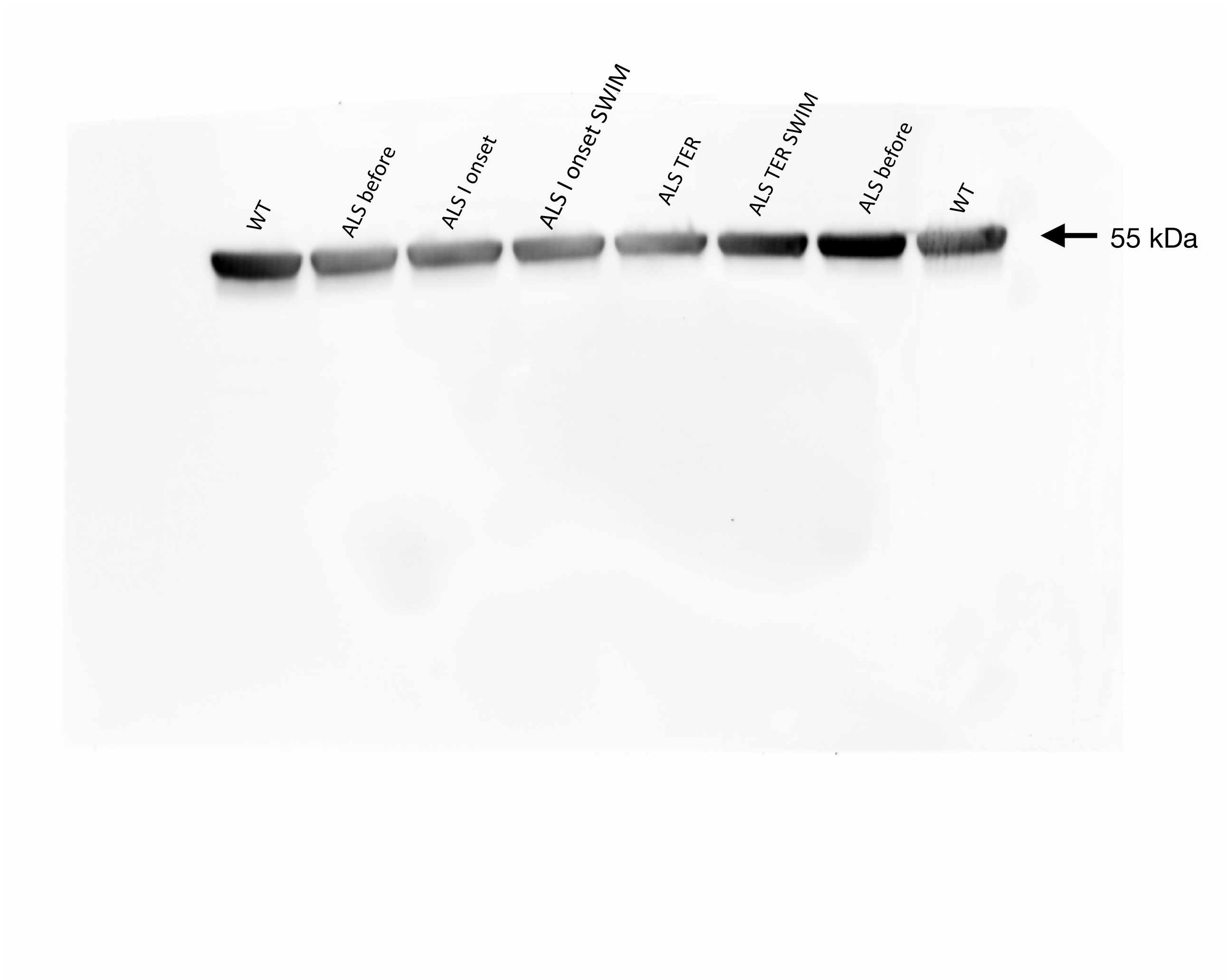
WT
ALS before
ALS I onset
ALS I onset SWIM
ALS TER
ALS TER SWIM
ALS before
WT
55 kDa

Stain free + protein marker for β-tubulin

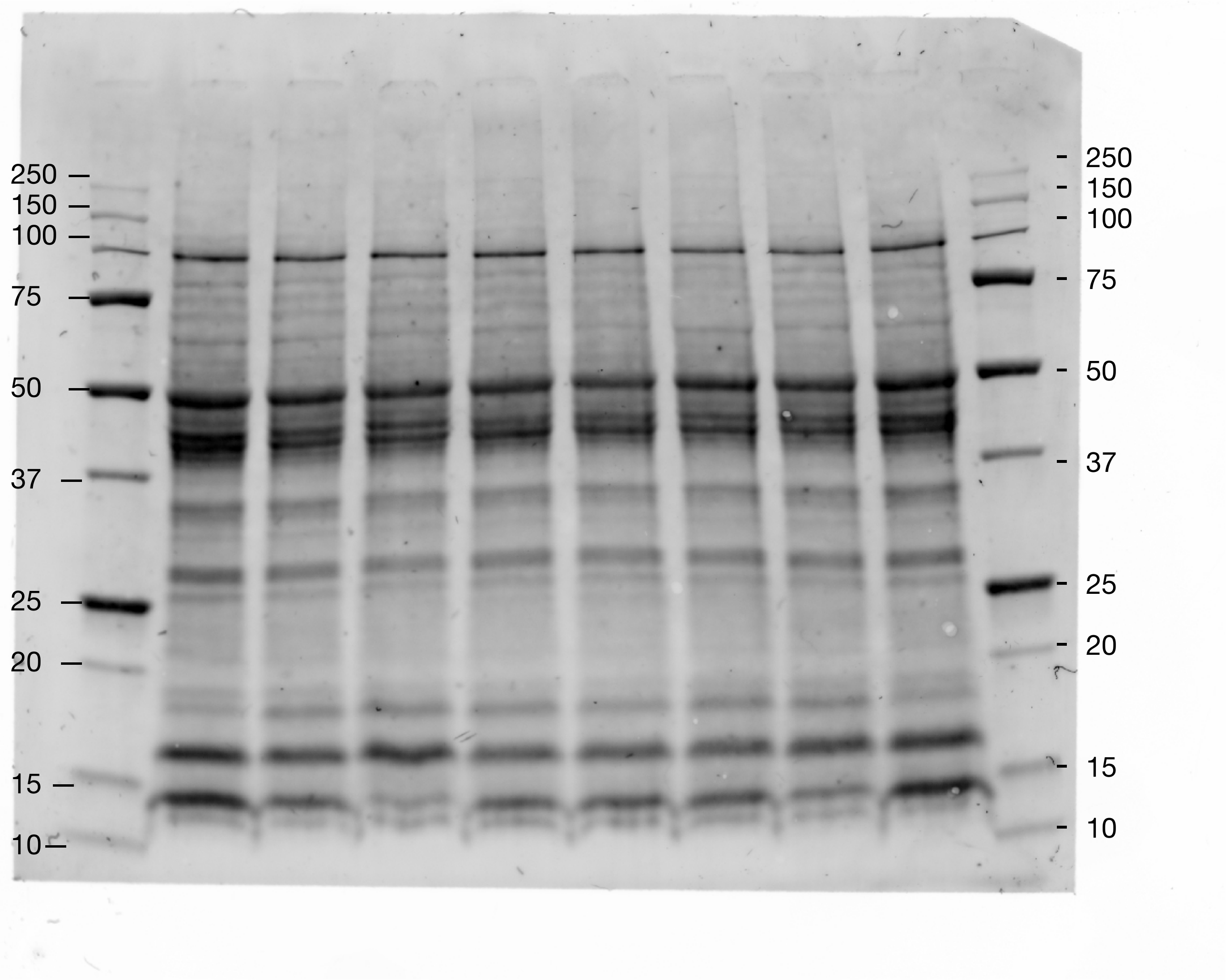

Supplement: Supplementary file 2 — Supplementary file2 (PDF 2.70 MB) [file 109_2023_2410_MOESM2_ESM.pdf]
