# Supplementary material for: Spinal cord abnormal autophagy and mitochondria energy metabolism are modified by swim training in SOD1-G93A mice
Source: J Mol Med (Berl). 2024 Jan 10;102(3):379–90. doi: 10.1007/s00109-023-02410-8 (PMC10879285; doi:10.1007/s00109-023-02410-8)

Supplementary data.
Full unedited versions of the western blots for Figure 3.

Nf-l

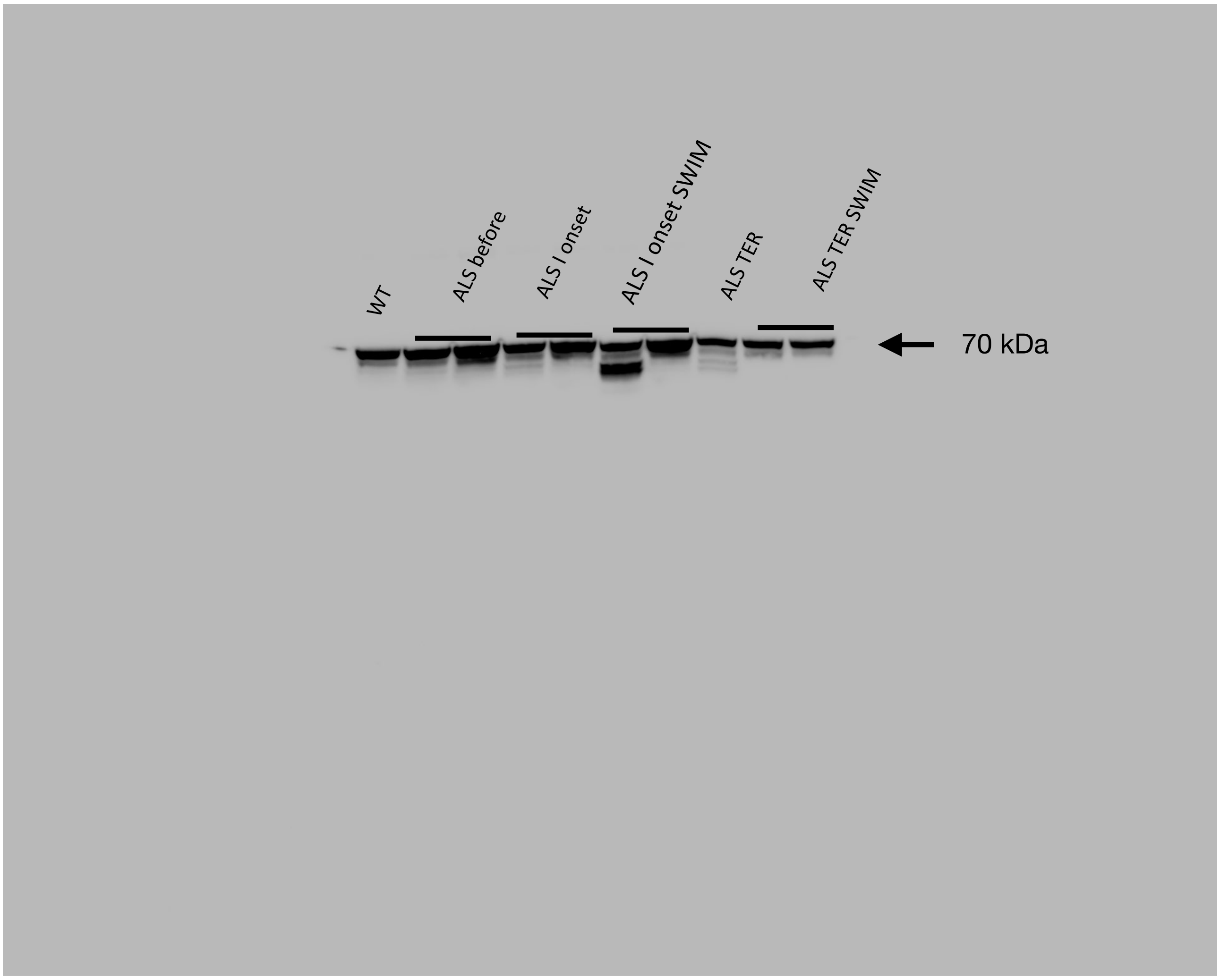

Stain free + protein marker for Nf-l

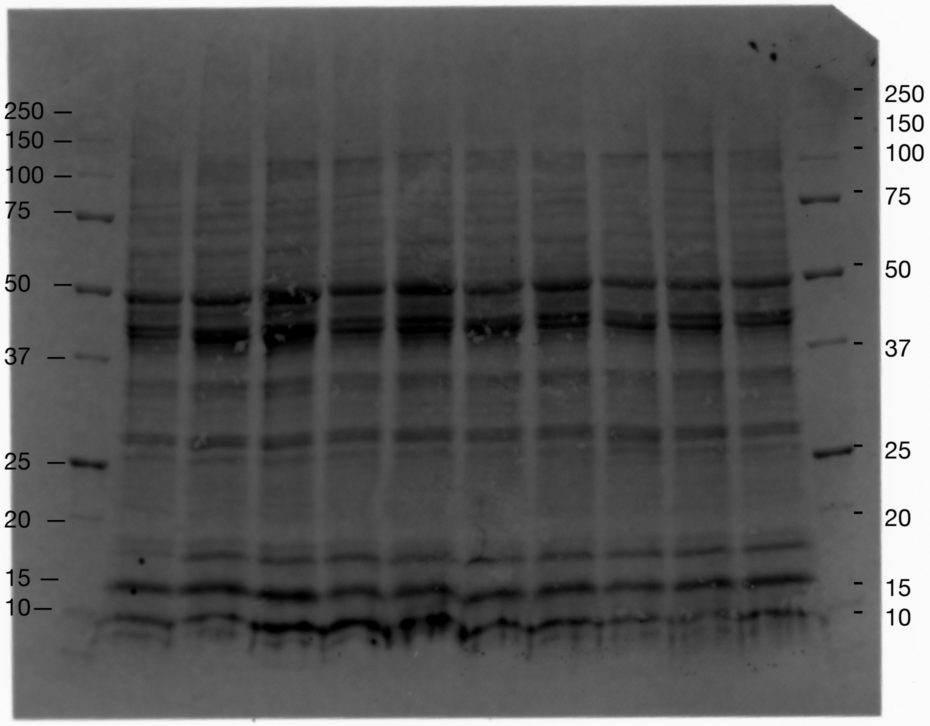

TBK-1

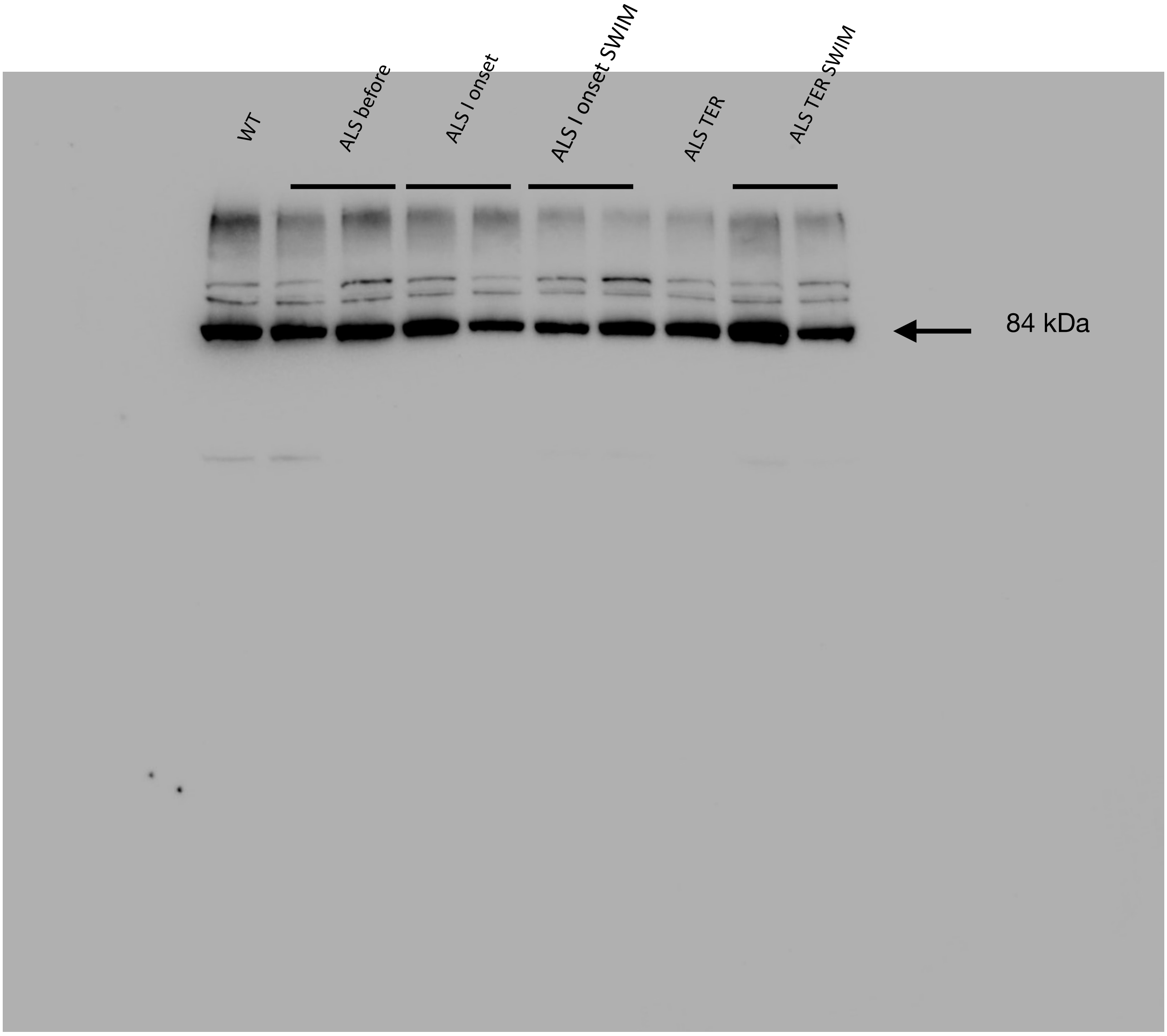

WT
ALS before
ALS I onset
ALS I onset SWIM
ALS TER
ALS TER SWIM
84 kDa

Stain free + protein marker for TBK1

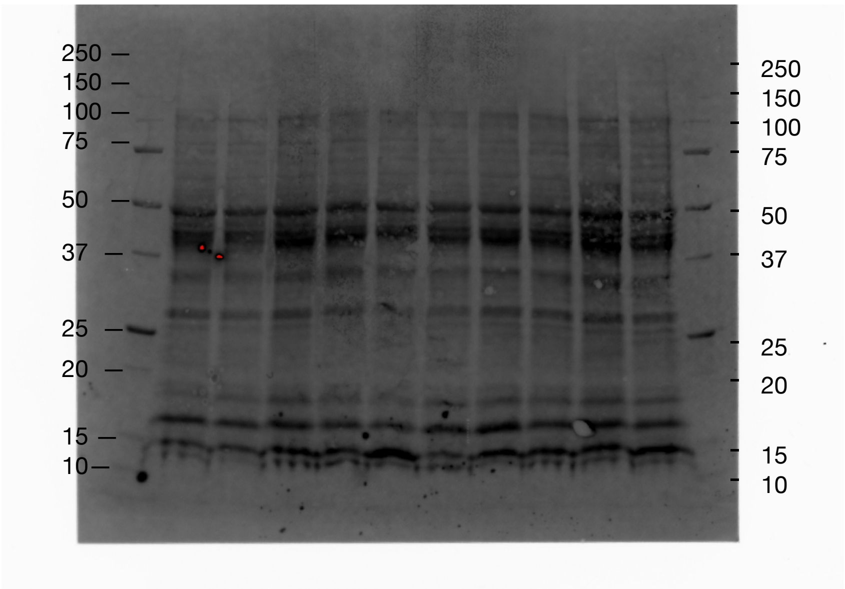

IGF-1

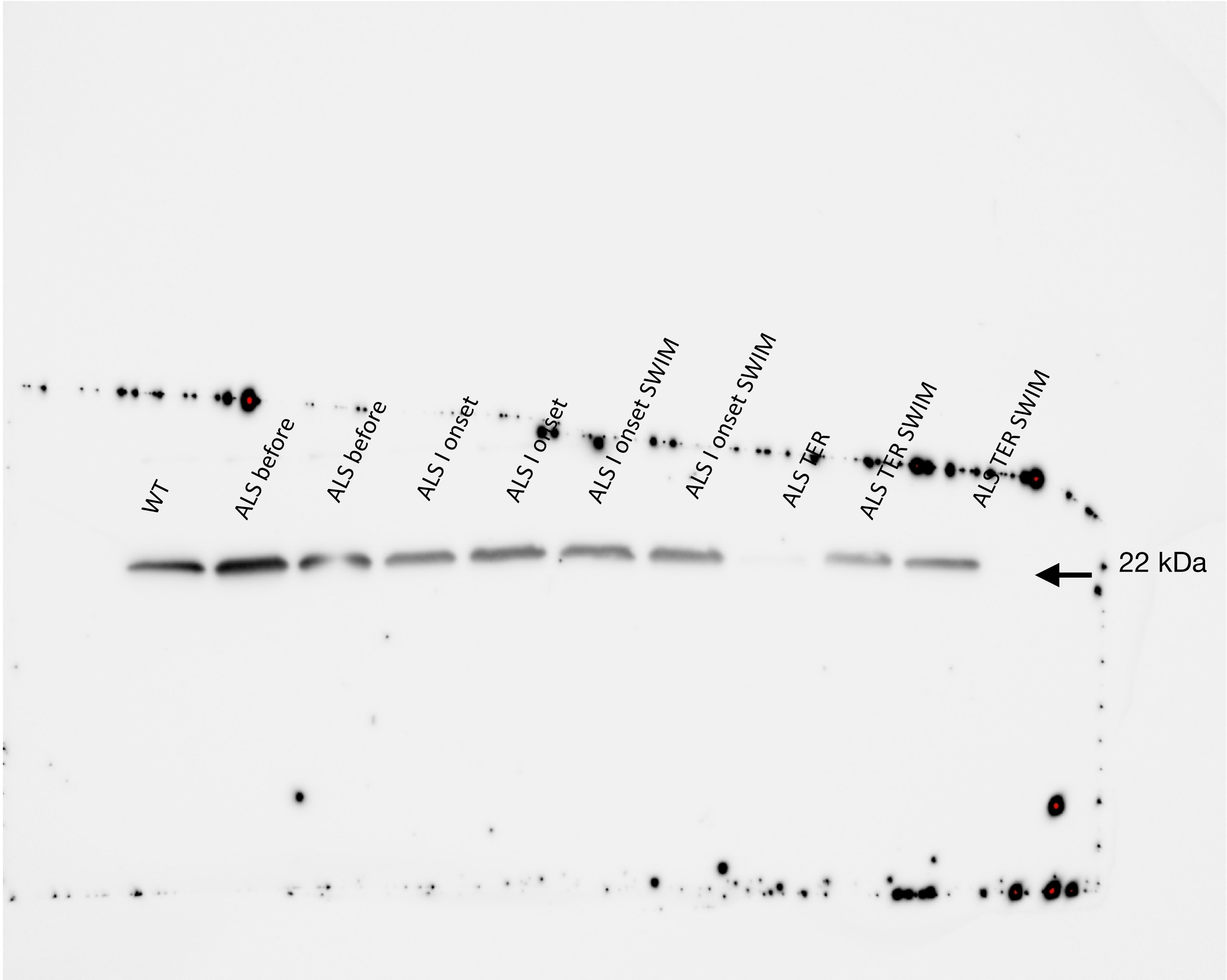
WT
ALS before
ALS before
ALS I onset
ALS I onset
ALS I onset SWIM
ALS I onset SWIM
ALS TER
ALS TER SWIM
ALS TER SWIM
22 kDa

Stain free + protein marker for IGF-1

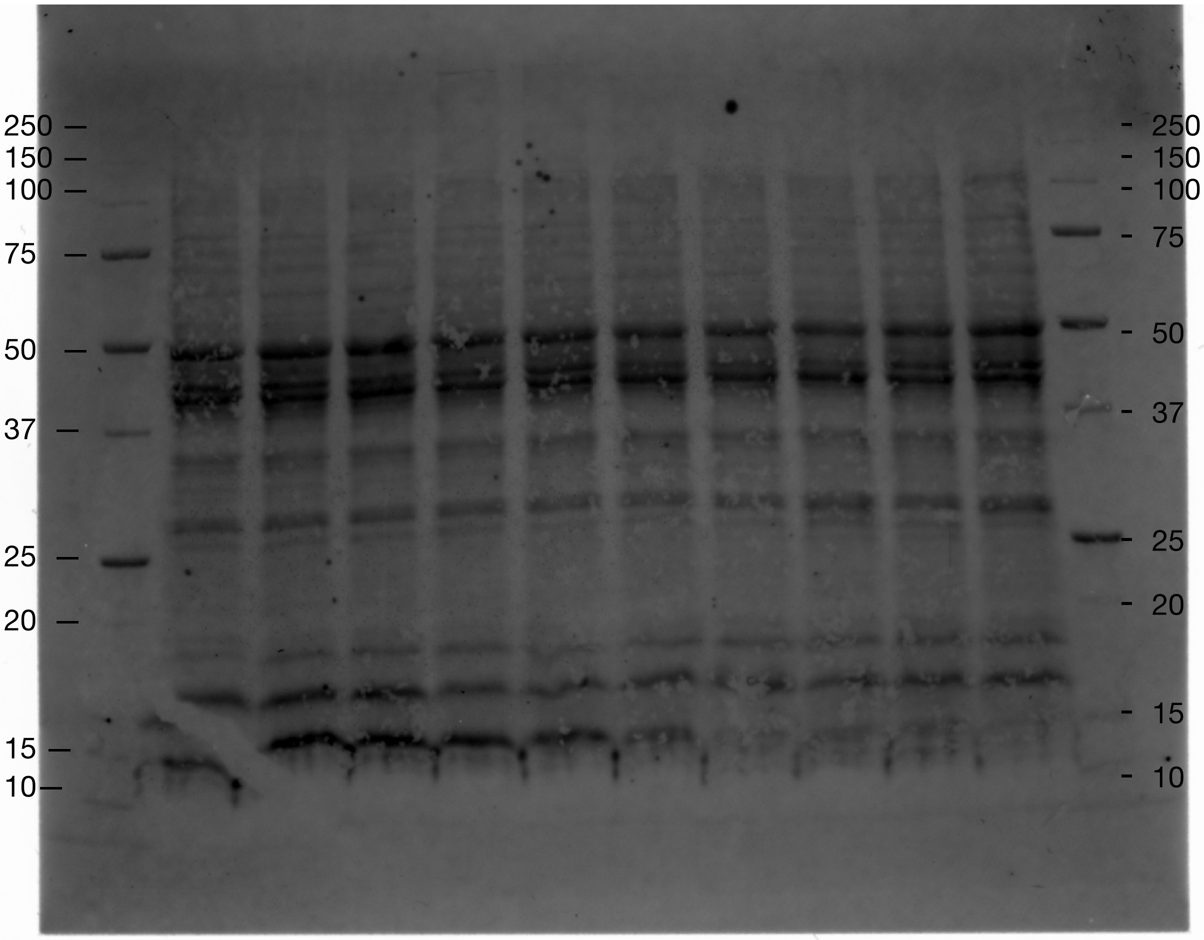

PGC-1α

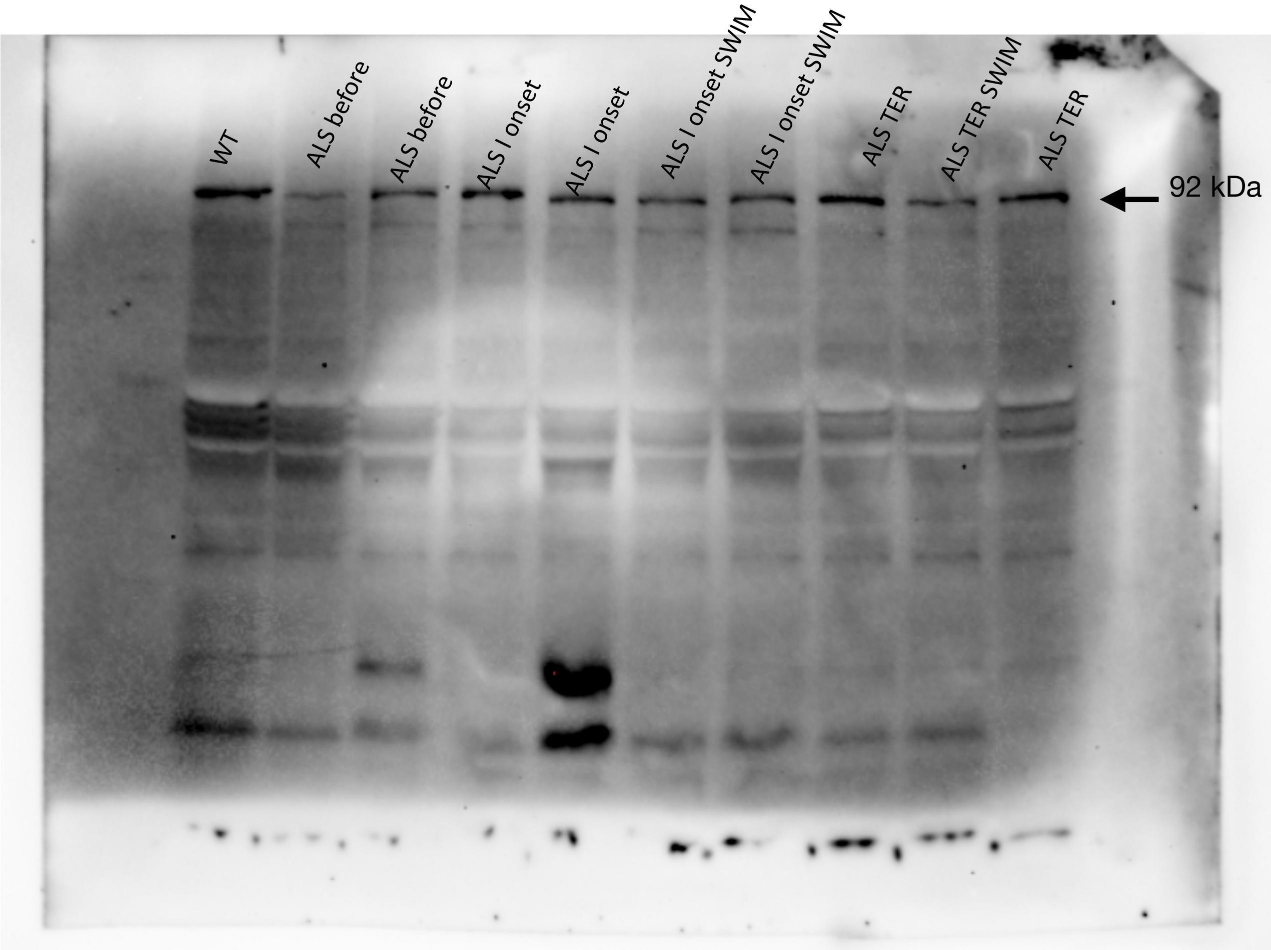
WT
ALS before
ALS before
ALS I onset
ALS I onset
ALS I onset SWIM
ALS I onset SWIM
ALS TER
ALS TER SWIM
ALS TER
92 kDa

Stain free + protein marker for PGC-1α

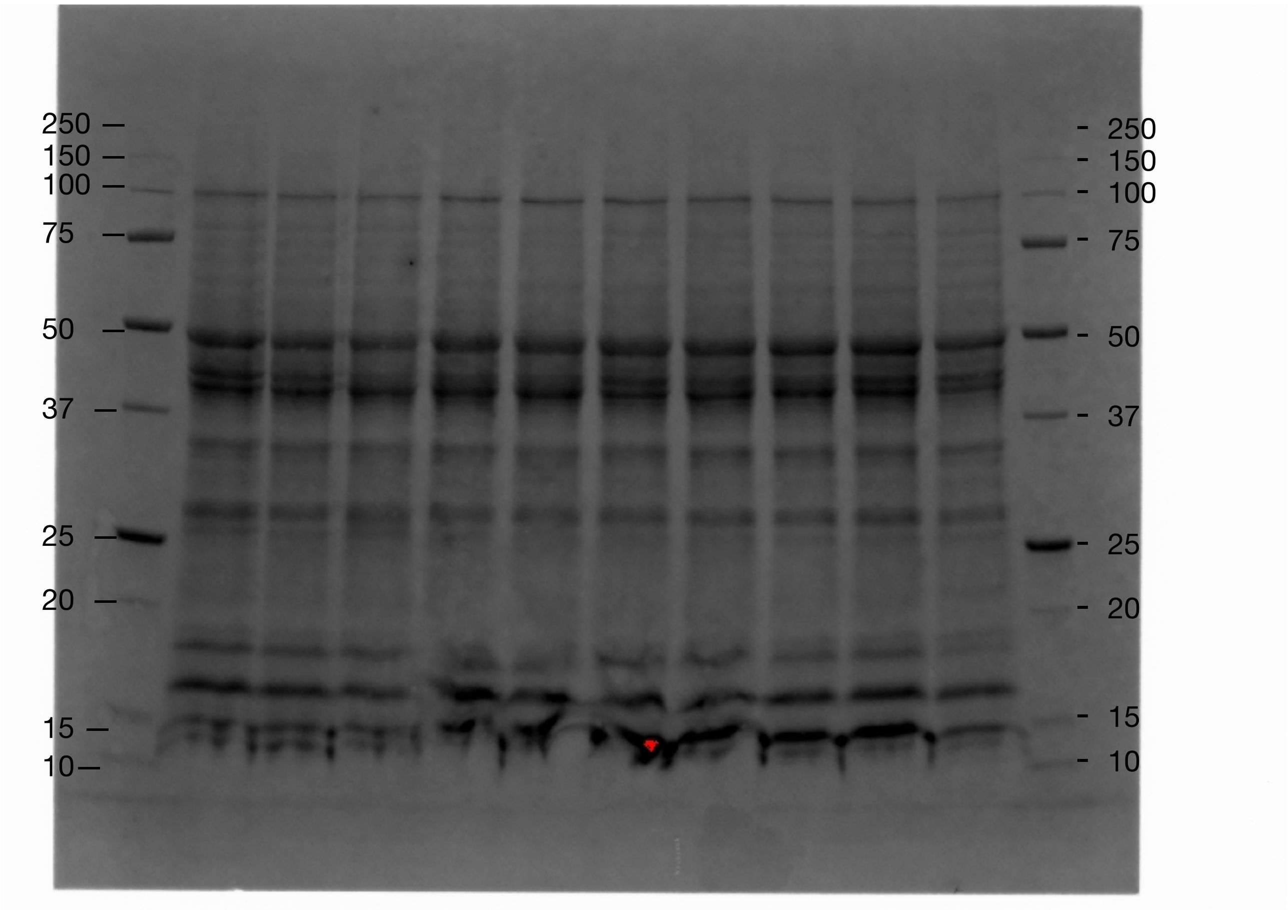

β-tubulin

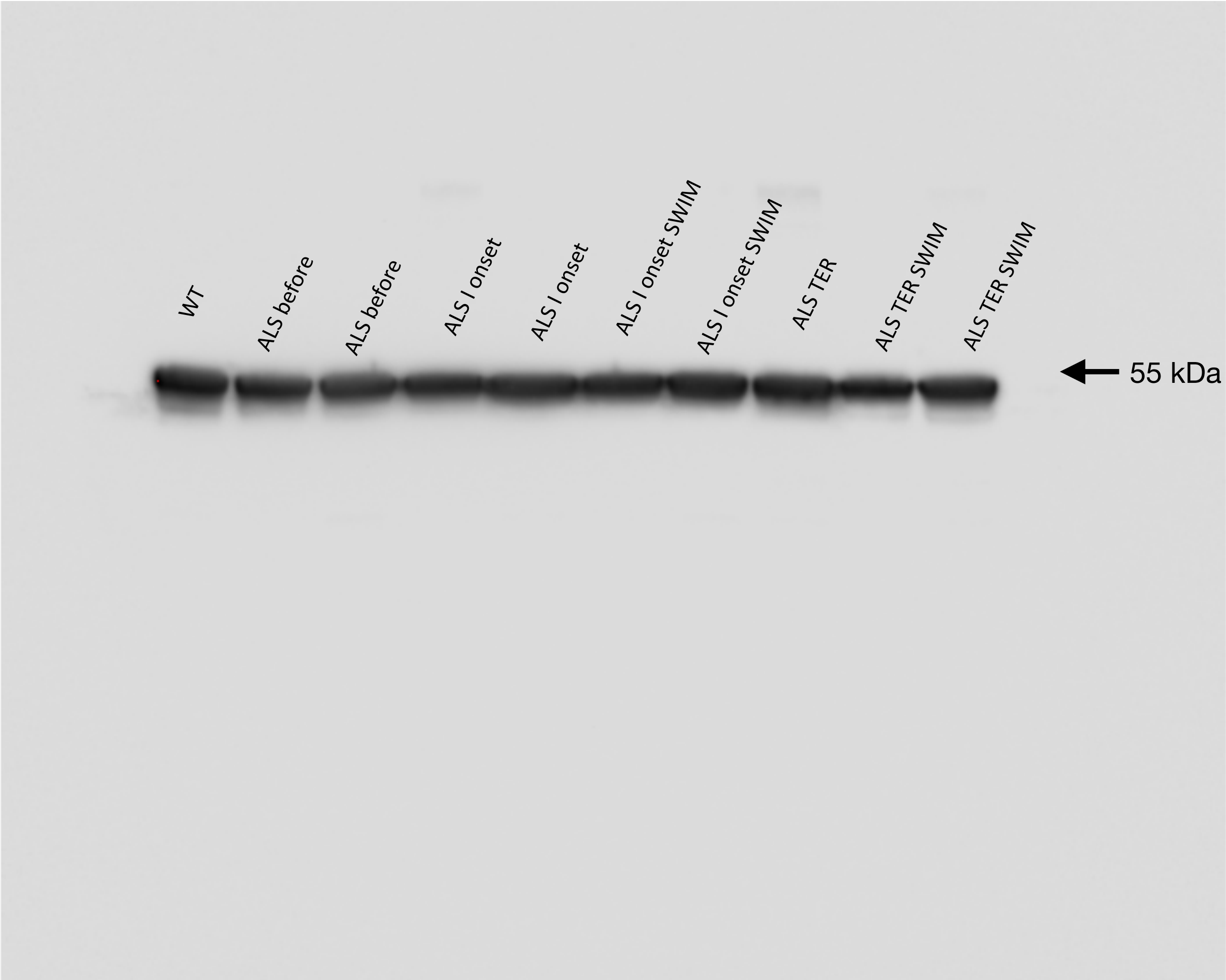
WT
ALS before
ALS before
ALS I onset
ALS I onset
ALS I onset SWIM
ALS I onset SWIM
ALS TER
ALS TER SWIM
ALS TER SWIM
55 kDa

Stain free + protein marker for β-tubulin

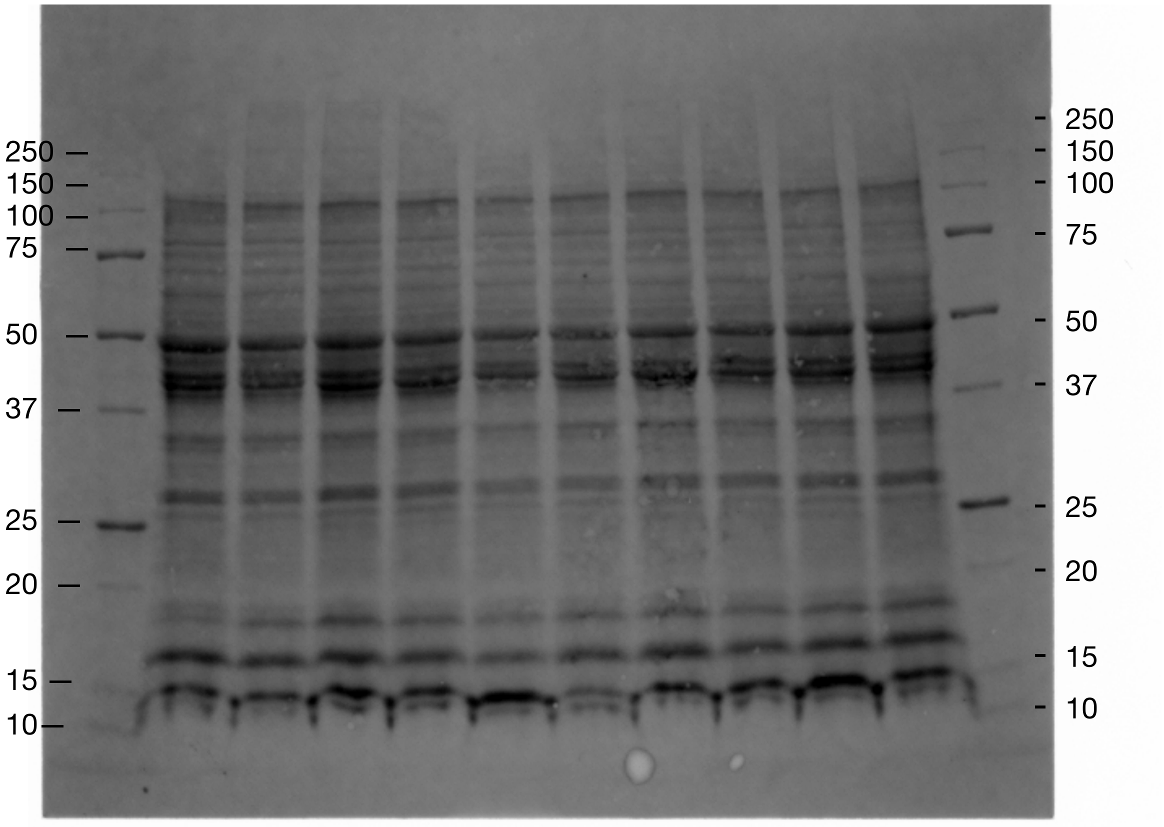

Supplement: Supplementary file 3 — Supplementary file3 (PDF 2.62 MB) [file 109_2023_2410_MOESM3_ESM.pdf]
