# Supplementary material for: Spinal cord abnormal autophagy and mitochondria energy metabolism are modified by swim training in SOD1-G93A mice
Source: J Mol Med (Berl). 2024 Jan 10;102(3):379–90. doi: 10.1007/s00109-023-02410-8 (PMC10879285; doi:10.1007/s00109-023-02410-8)

Supplementary data.
Full unedited versions of the western blots for Figure 4.

COX IV

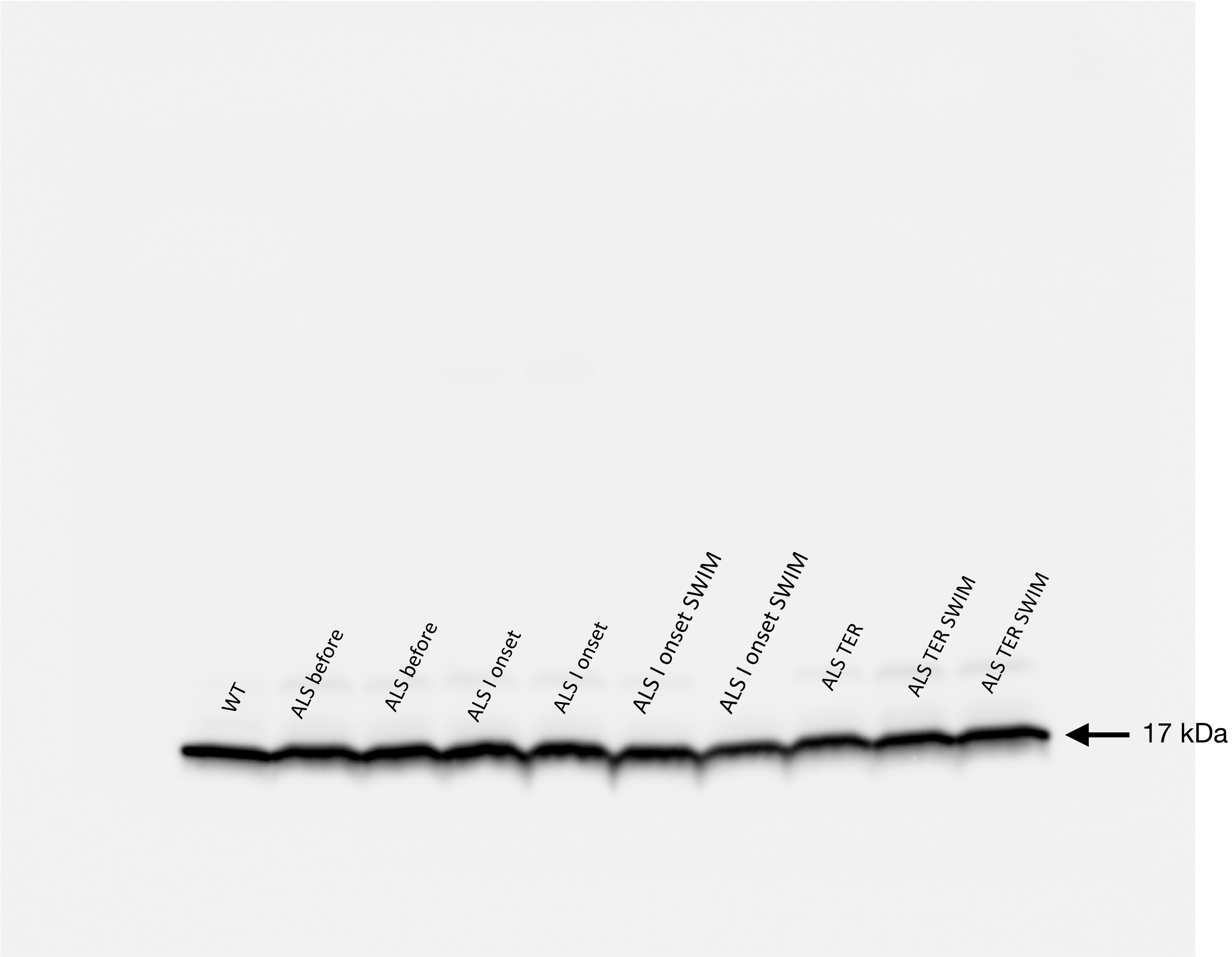

Stain free + protein marker for COX IV

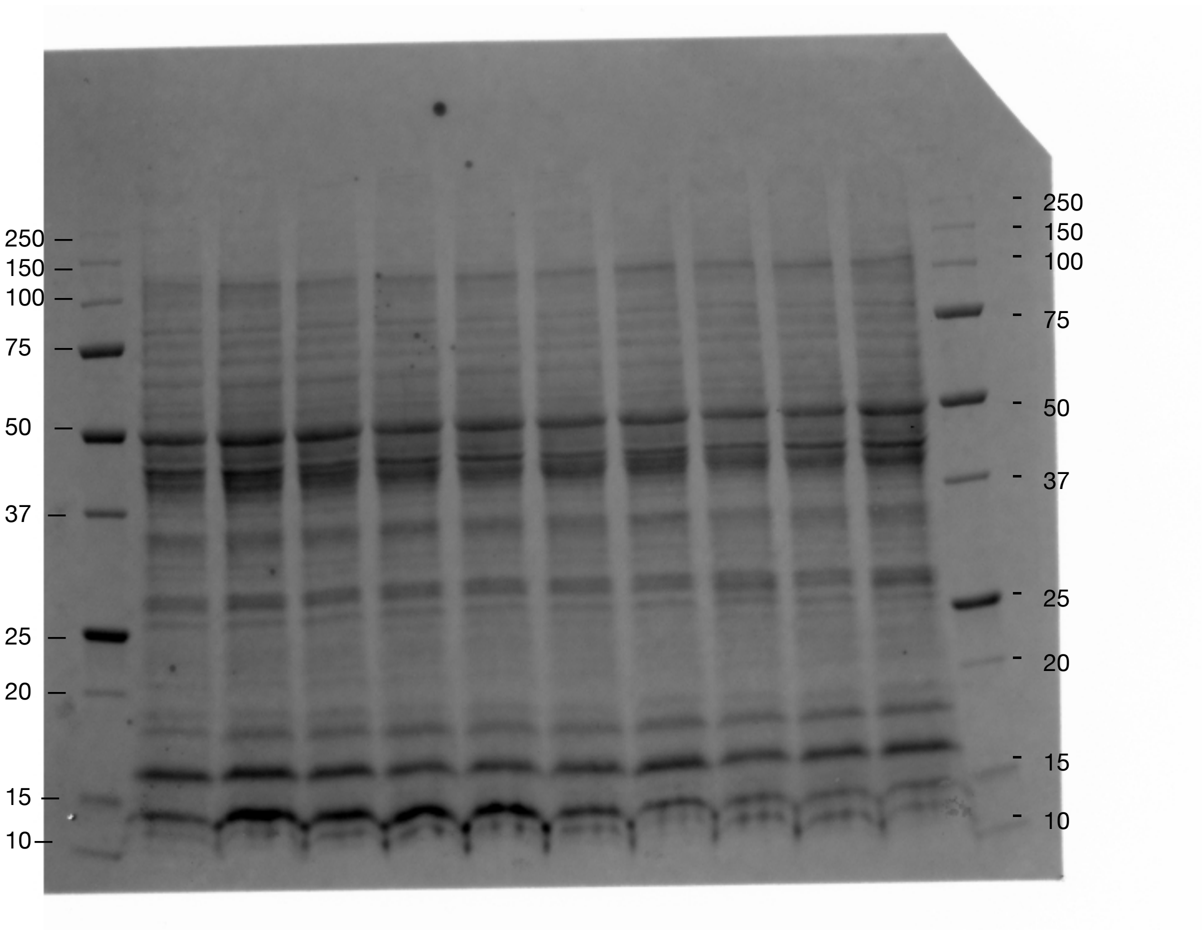

COX II

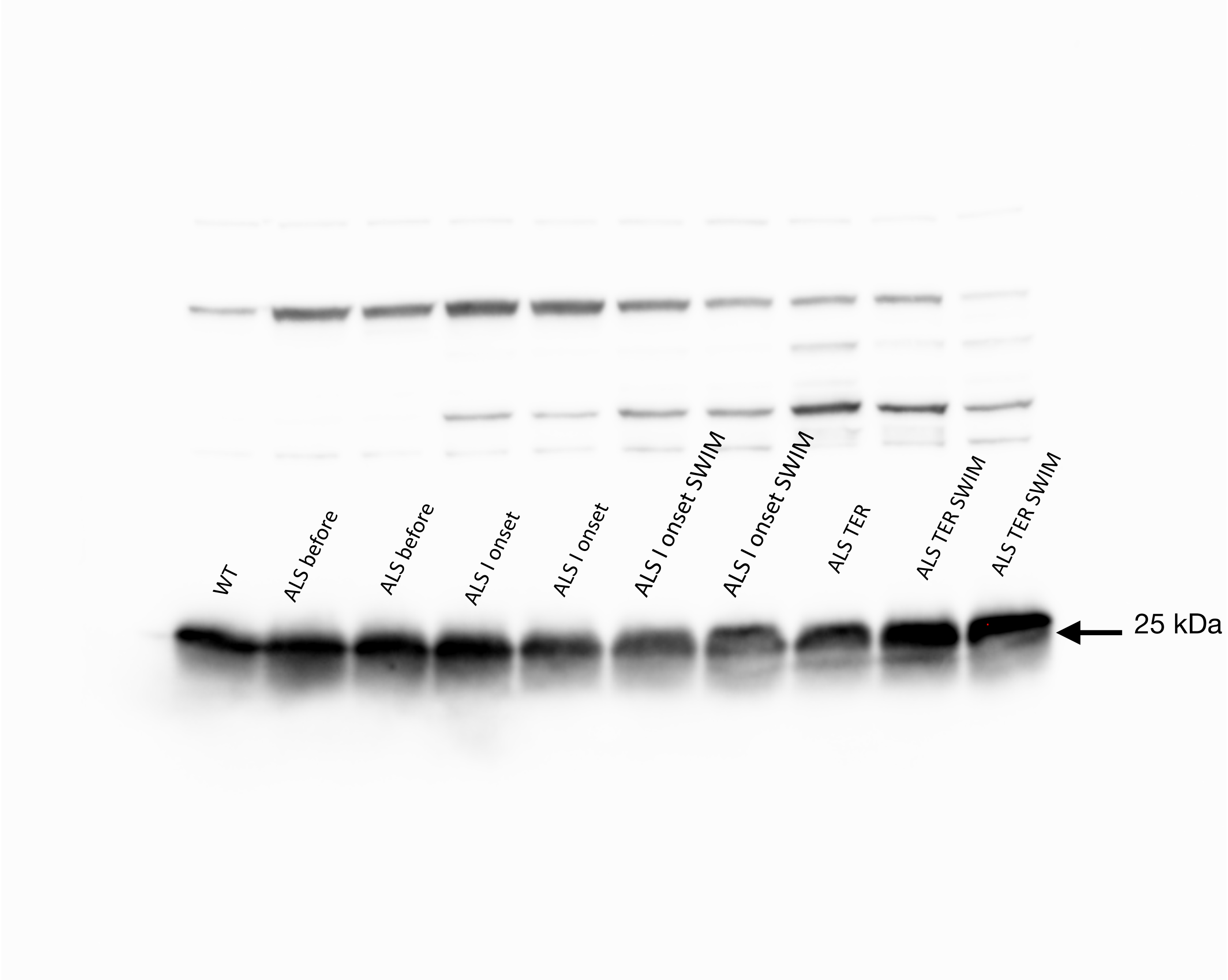
WT
ALS before
ALS before
ALS I onset
ALS I onset
ALS I onset SWIM
ALS I onset SWIM
ALS TER
ALS TER SWIM
ALS TER SWIM
25 kDa

Stain free + protein marker for COX II

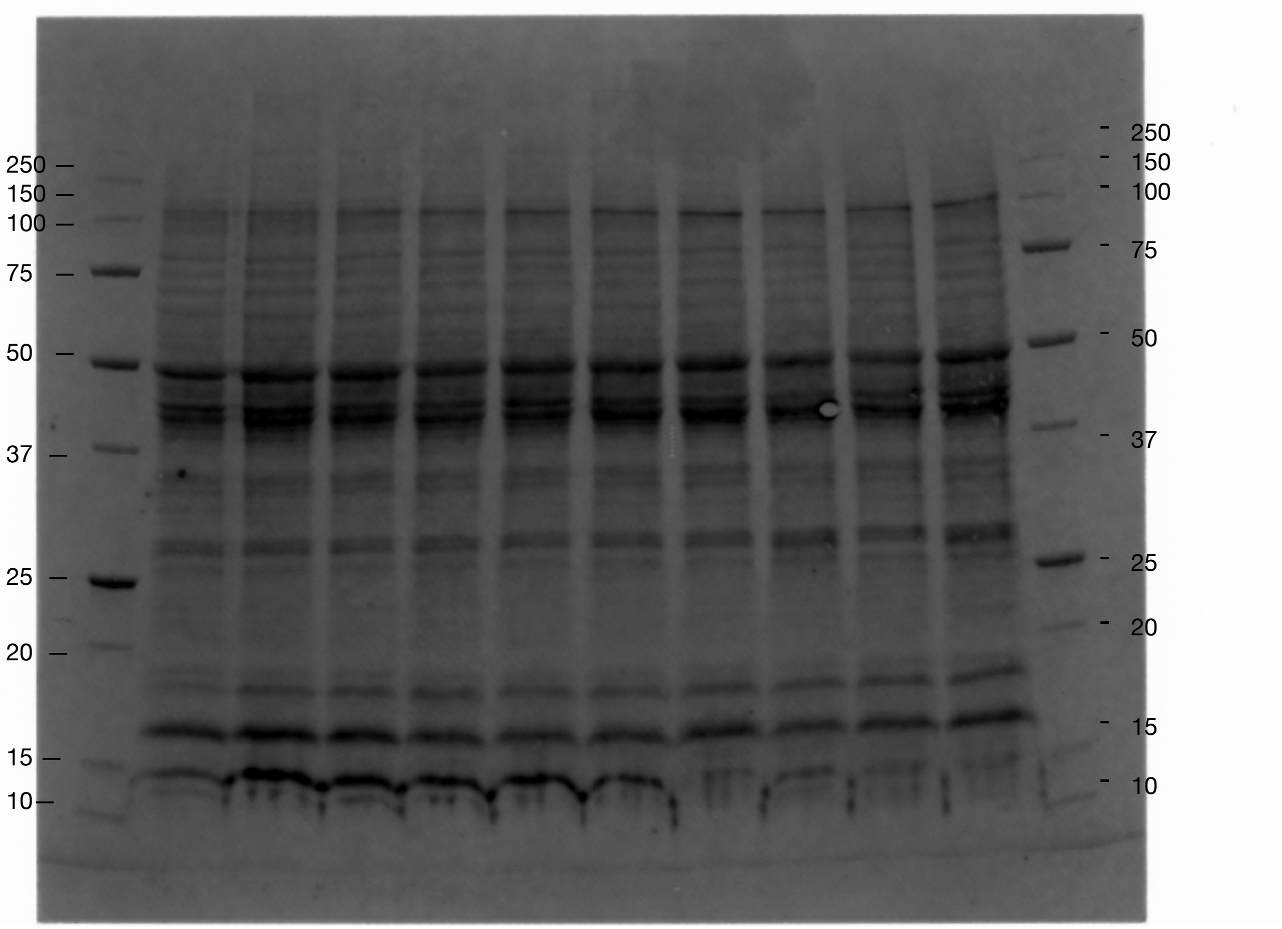

SCO1

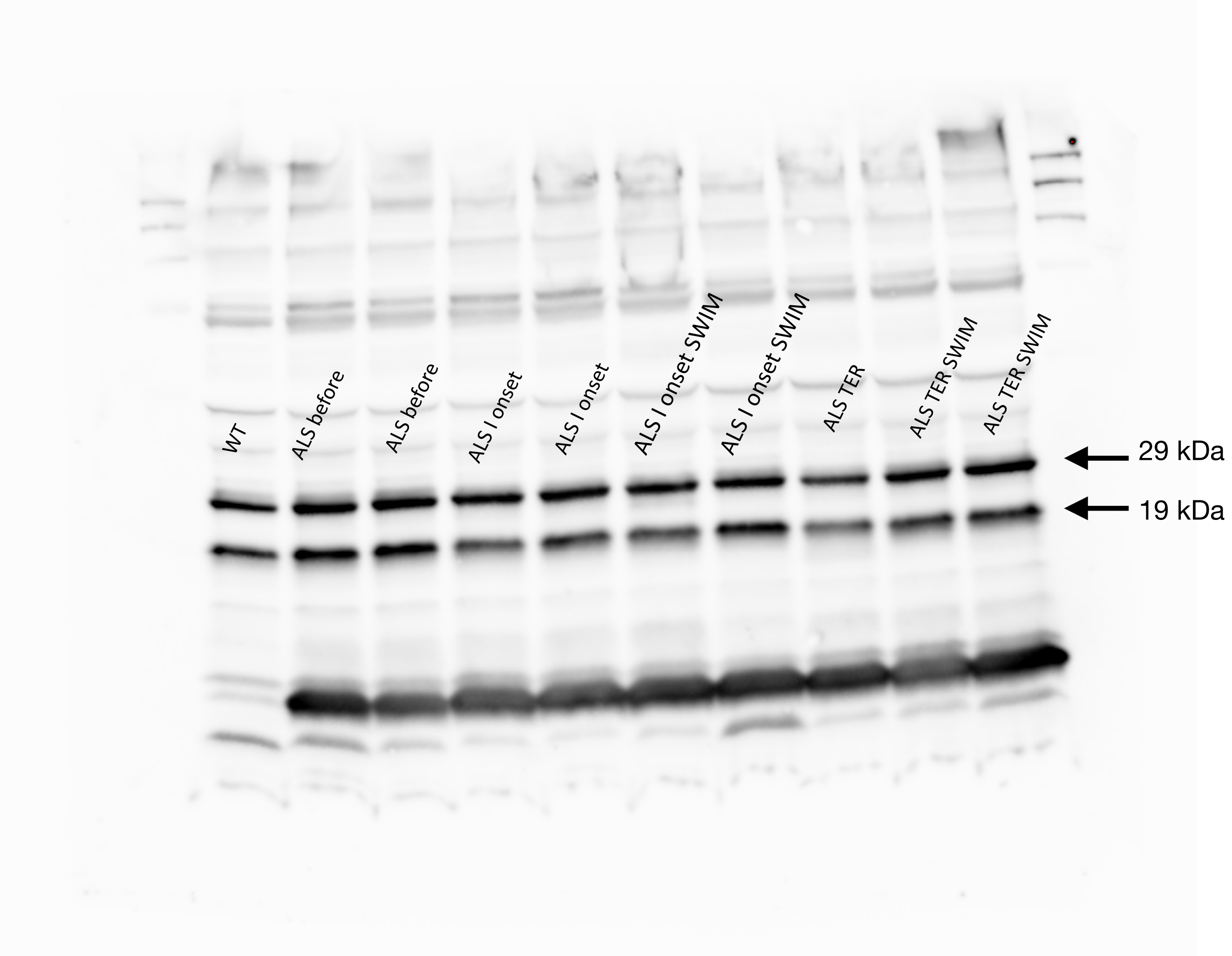

Stain free + protein marker for SCO1

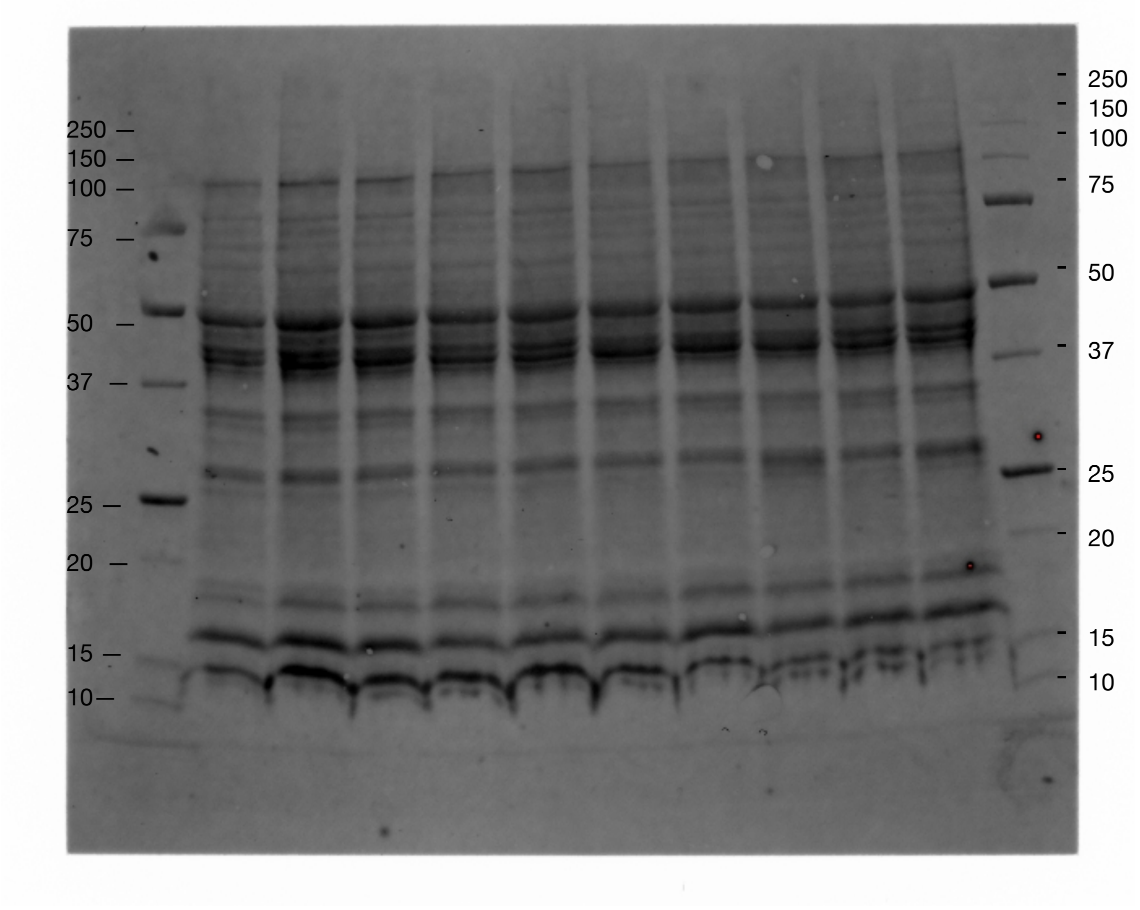

SCO2

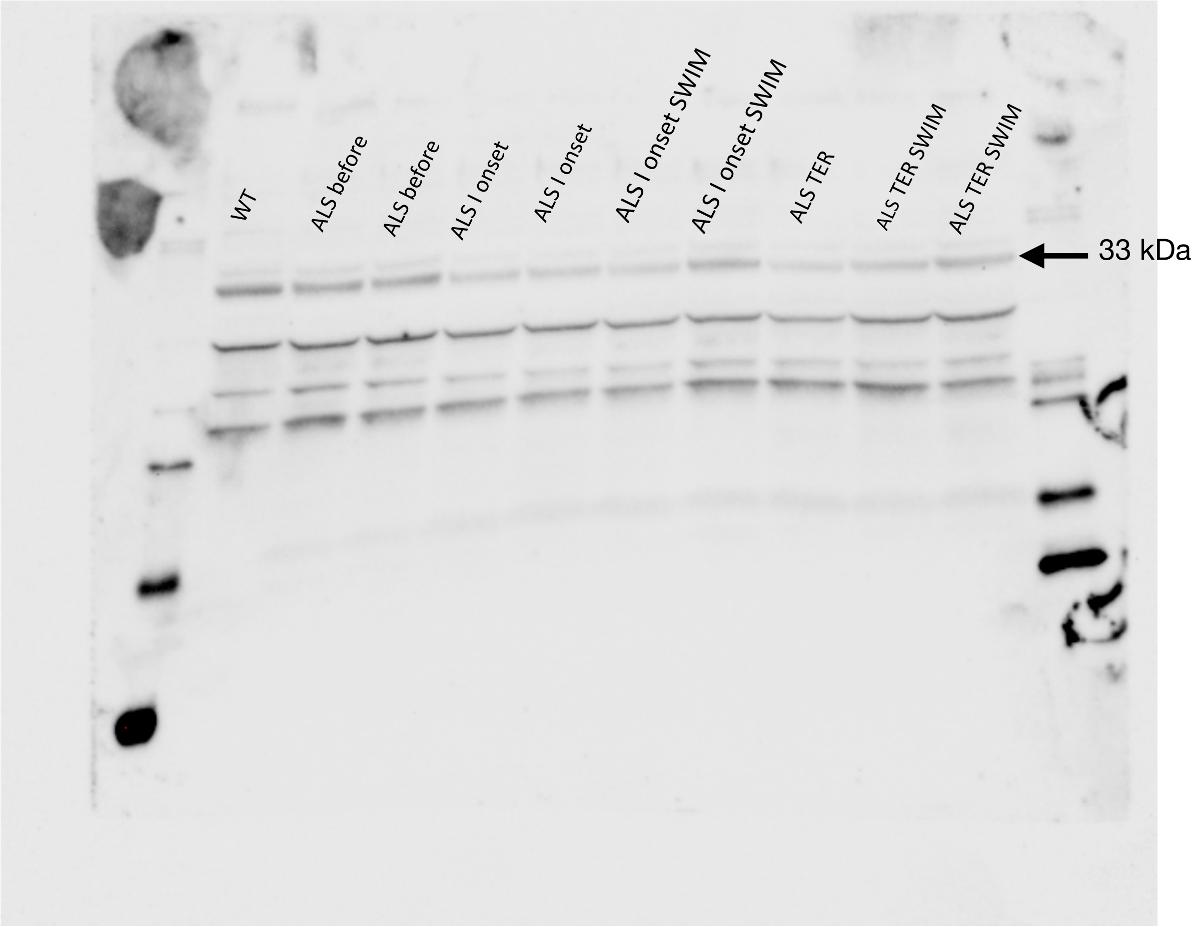

WT
ALS before
ALS before
ALS I onset
ALS I onset
ALS I onset SWIM
ALS I onset SWIM
ALS TER
ALS TER SWIM
ALS TER SWIM
33 kDa

Stain free + protein marker for SCO2,

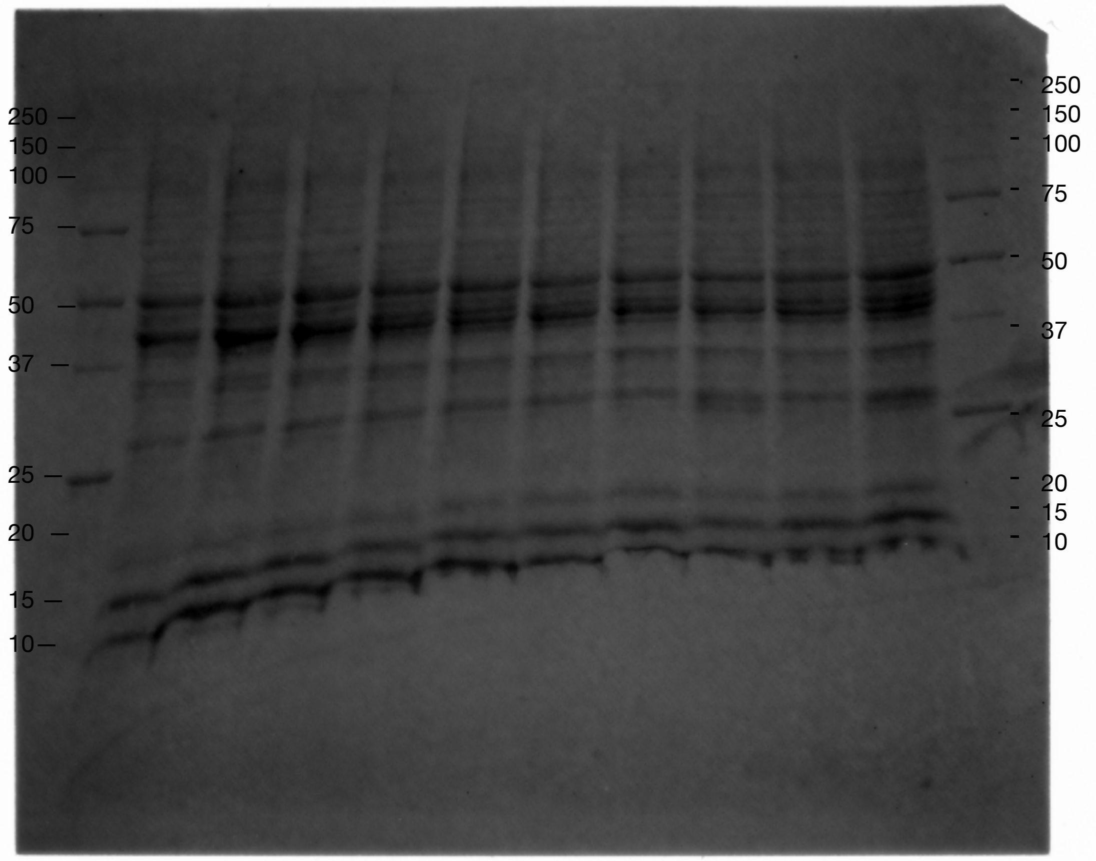

OGDH, membrane cut from IGF-1

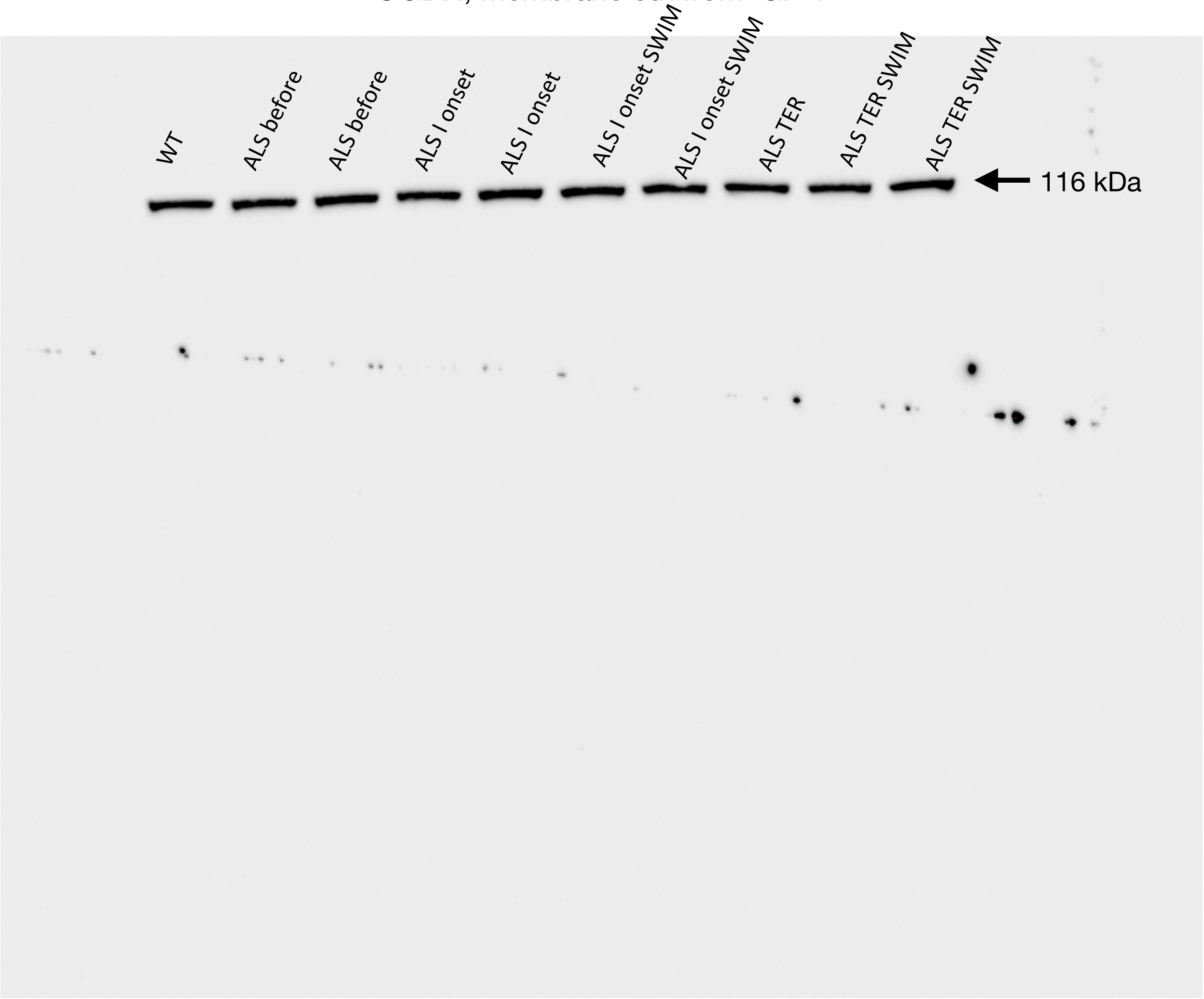

Stain free + protein marker for OGDH

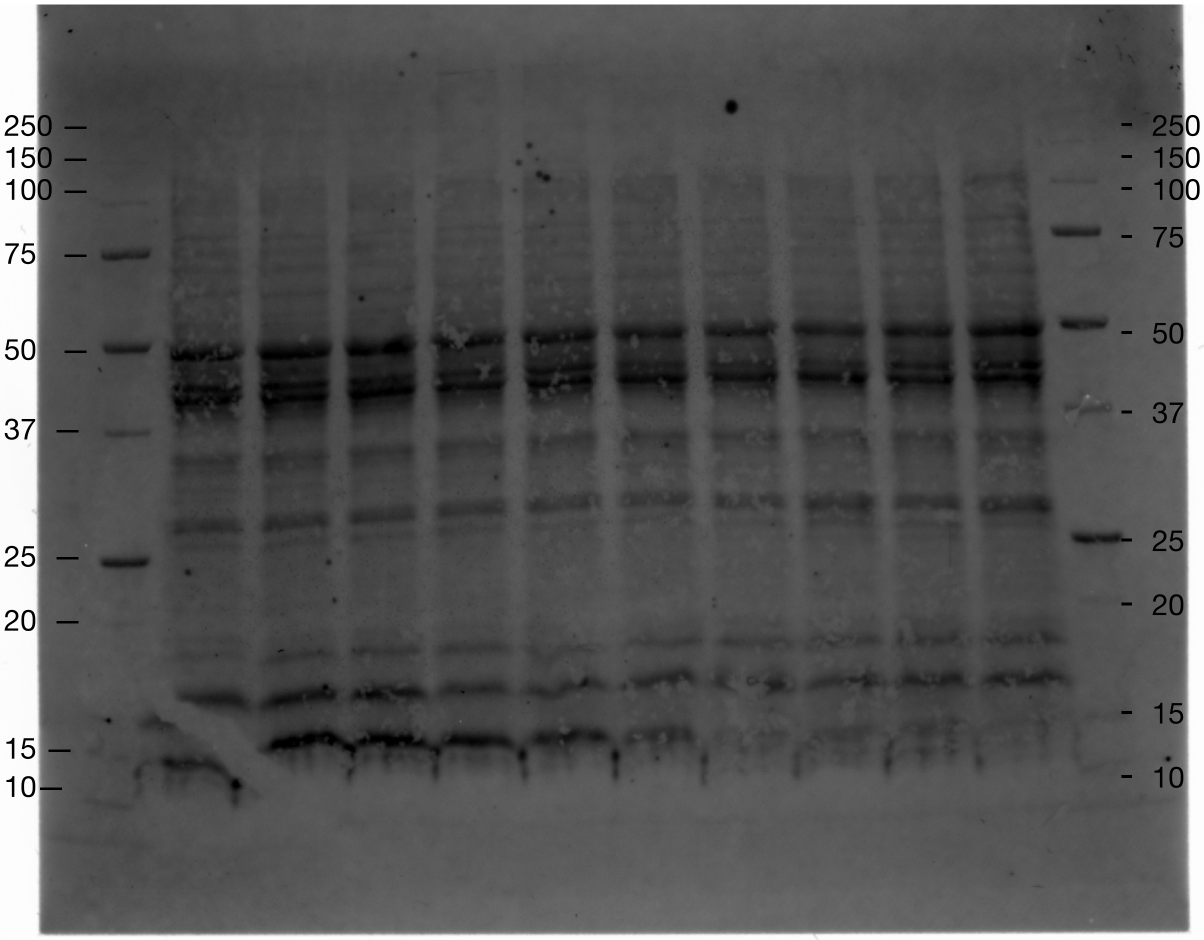

β-tubulin

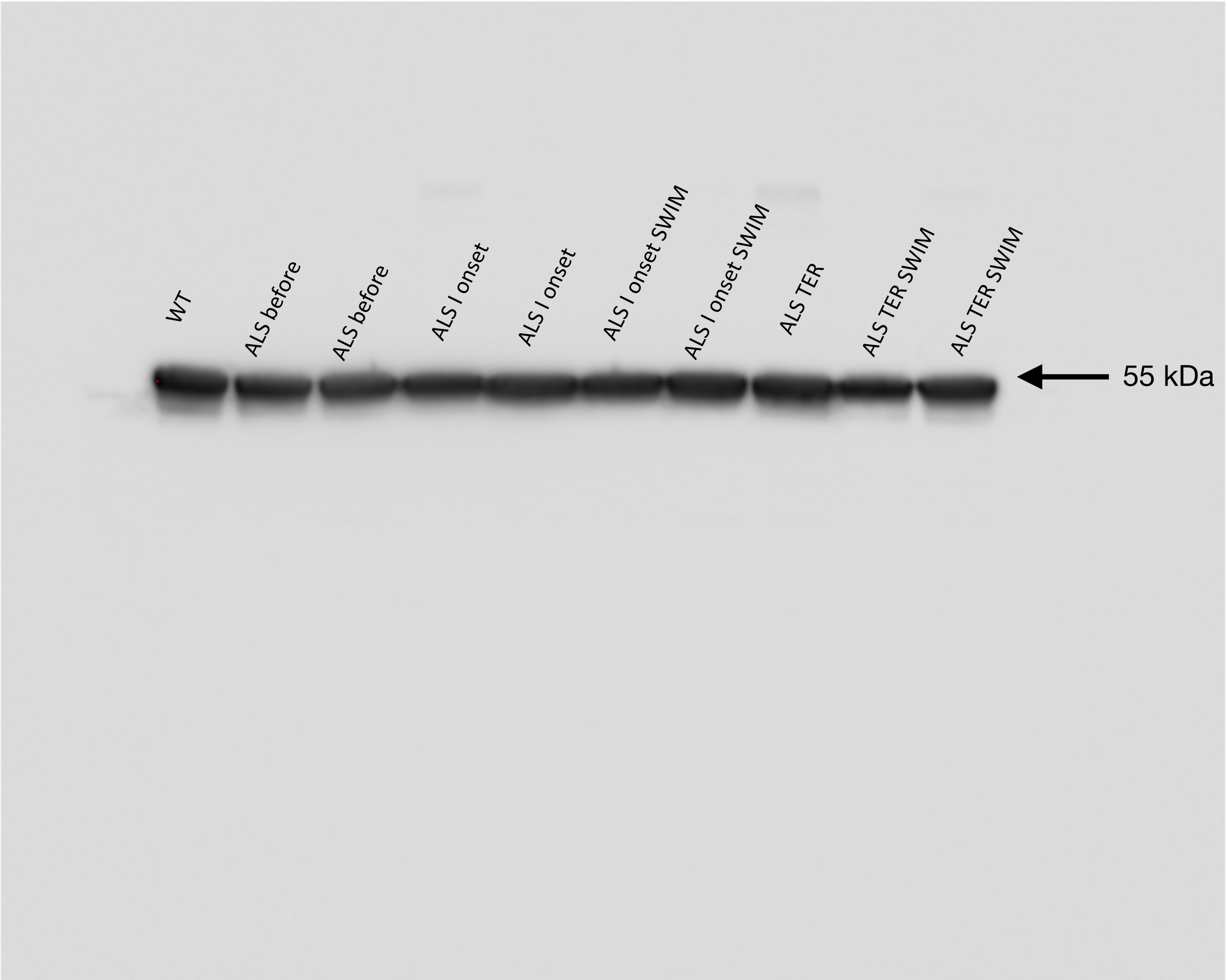
WT
ALS before
ALS before
ALS I onset
ALS I onset
ALS I onset SWIM
ALS I onset SWIM
ALS TER
ALS TER SWIM
ALS TER SWIM
55 kDa

Stain free + protein marker for β-tubulin

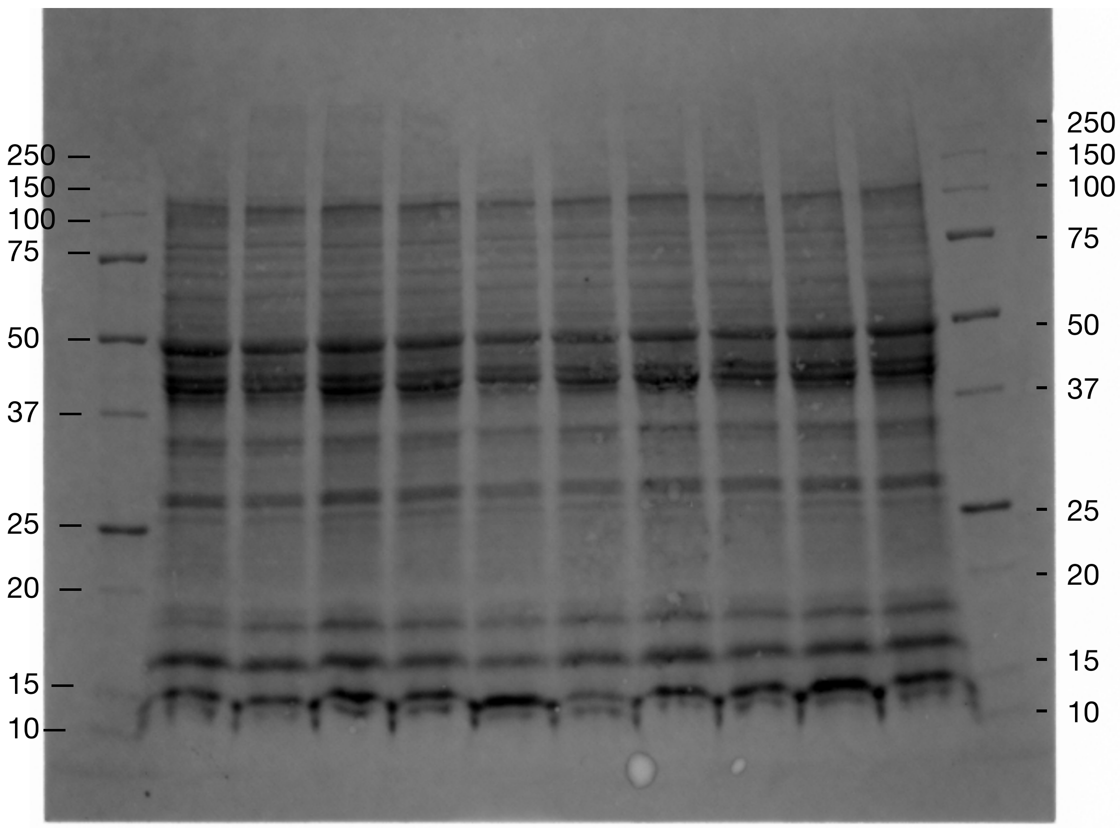

Supplement: Supplementary file 4 — Supplementary file4 (PDF 4.57 MB) [file 109_2023_2410_MOESM4_ESM.pdf]
